# Quantitative Assessment of Total Aerobic Viable Counts in Apitoxin-, Royal-Jelly-, Propolis-, Honey-, and Bee-Pollen-Based Products Through an Automated Growth-Based System

**DOI:** 10.3390/microorganisms14010218

**Published:** 2026-01-17

**Authors:** Harold A. Prada-Ramírez, Raquel Gómez-Pliego, Humberto Zardo, Willy-Fernando Cely-Veloza, Ericsson Coy-Barrera, Rodrigo Palacio-Beltrán, Romel Peña-Romero, Sandra Gonzalez-Alarcon, Juan Camilo Fonseca-Acevedo, Juan Pablo Montes-Tamara, Lina Nieto-Celis, Ruth Dallos-Acosta, Tatiana Gonzalez, David Díaz-Báez, Gloria Inés Lafaurie

**Affiliations:** 1 Laboratorios Coaspharma S.A.S., CL 18A 28A-43, Bogotá 111411, Colombia; rodrigo.palacios@coaspharma.co (R.P.-B.); romel.pena@coaspharma.co (R.P.-R.); sandra.gonzalez@coaspharma.co (S.G.-A.); camilo.fonseca@coaspharma.co (J.C.F.-A.); juan.montes@coaspharma.co (J.P.M.-T.); lina.nieto@coaspharma.co (L.N.-C.); 2 Departamento de Ciencias Biológicas, Sección de Ciencias de la Salud Humana, Facultad de Estudios Superiores Cuautitlán-UNAM, Cuautitlán Izcalli 54740, Estado de México, Mexico; pliegoraquel@comunidad.unam.mx; 3 School of Pharmaceutical Sciences, University of São Paulo, São Paulo 05508-000, SP, Brazil; huzardo@yahoo.com; 4 Unidad de Investigación Básica Oral—UIBO, Vicerrectoría de Investigaciones, Facultad de Odontología, Universidad El Bosque, Bogotá 111321, Colombia; willy.cely@unimilitar.edu.co (W.-F.C.-V.); david.diazb@upb.edu.co (D.D.-B.); 5 Laboratorio de Química Bioorgánica, Facultad de Ciencias Básicas y Aplicadas, Universidad Militar Nueva Granada, Cajicá 250247, Colombia; ericsson.coy@unimilitar.edu.co; 6 Neogen Colombia S.A.S., Bogotá 0810, Colombia; rdallos@neogen.com (R.D.-A.); tgonzalez@neogen.com (T.G.)

**Keywords:** apitoxin, royal jelly, propolis, bee pollen, honey, bacteria, yeast, molds, automated growth-based system

## Abstract

Bee-derived products such as apitoxin, royal jelly, propolis, bee pollen, and honey are increasingly being used as part of cosmetic products because all of them contain a large number of bioactive compounds with antioxidant, anti-inflammatory, antimicrobial, and regenerative properties, which enable them to be used for therapeutic purposes. The aim of this investigation was to assess the performance of an automated growth-based system in order to make a quantitative examination of the total aerobic viable counts in bee-derived personal care products using NF-TVC vials that contained a nutrient-based medium with dextrose as the carbon source. According to USP general chapter <1223>, pivotal validation criteria such as linearity, equivalence of results, operative range, precision, accuracy, ruggedness, limit of quantification, and limit of detection have demonstrated that the automated system can be used for a reliable total aerobic viable count. Moreover, the actual research demonstrated that polysorbates efficiently block the antimicrobiological potential of bioactive compounds, such as phenols, flavonoids, enzymes, peptides, and fatty acids, which naturally occur in apitoxin, royal jelly, propolis, bee pollen, and honey, allowing for efficient microorganism recovery from the bee-made products tested. Therefore, this AGBS could be applied efficiently within the cosmetic industry to assess the total aerobic viable count in bee-derived products such as capillary treatments, toothpaste, and anti-aging cream, affording several benefits associated with faster product release into the market.

## 1. Introduction

Natural products such as apitoxin, royal jelly, propolis, bee pollen, and honey are increasingly being used in the cosmetic industry because their broad spectrum of bioactive compounds, such as phenols, flavonoids, enzymes, proteins, and fatty acids, have wound-healing properties, high nutritional value, and regenerative potential that enable them to be included in several personal care formulations as cosmetic products [1,2,3]. These biological compounds are active in combating oxidative stress, promoting collagen production, reducing inflammation, minimizing facial line expression, and stimulating capillary cellular regeneration [3,4,5,6,7,8,9,10,11,12]. One significant challenge in the cosmetic and pharmaceutical industries is the prolonged delay caused by microbiological quality control since the traditional methods for total aerobic viable count (TAVC) typically take up to 7 days. The present investigation aims to assess the performance of an automated growth-based system (AGBS) in examining the TAVC from bee-derived personal care products. This AGBS brings an array of benefits, such as reducing the microbiological process time for TAVC from 7 days, as with the reference standard method (RSM), to just 2 days, with reliable outcomes since the AGBS meets all of the requirements outlined in general chapter <1223>, which are the suitability of the method, linearity, the equivalence of the results, operative range, precision, accuracy, robustness, specificity, limit of detection, and limit of quantification [12,13,14,15,16,17,18]. This substantial reduction in time is not only scientifically relevant but also highly impactful for the industry, as it enables faster product release, minimizes warehousing and inventory costs, enhances supply chain responsiveness, and ensures the timely detection of potential microbiological issues. Previously, we have demonstrated the good performance of the AGBS as a quantitative method using an antacid oral suspension as a pharmaceutical matrix to target objectionable pathogens, such as the *Burkholderia cepacia* complex, yeasts, and molds [12,13]. However, its performance using bee-made products such as apitoxin–royal-jelly-based anti-aging creams, propolis–honey-based toothpaste, and bee-pollen-, apitoxin-, and royal-jelly-based creams has been unexplored in terms of the quantitative TAVC. Regarding the antimicrobial properties of honey, apitoxin, royal jelly, bee pollen, and propolis, which prevent microbiological harvesting, it is interesting to test polysorbate as a chemical neutralizer of these bioactive compounds [1,2,3]. Therefore, tests of the method’s suitability were conducted for all bee-derived personal care products under study through chemical neutralization. The TAVC examination was performed by testing *Staphylococcus aureus*, *Escherichia coli*, *Pseudomonas aeruginosa*, *Candida albicans*, and *Aspergillus brasiliensis*, which are suitable representatives of aerobic viable microorganisms.

Regarding this matter, personal care products are not required to be completely aseptic; however, a rapid and accurate microbiological assessment is necessary to examine the microbial quality of the product and ensure compliance with microbiological specifications. Therefore, in microbiological specification terms, cosmetic products with bioregulator activity must have a TAVC <10^2^ CFU/mL or g., and harmful pathogens such as *S. aureus*, *P. aeruginosa*, and *E. coli* must be undetectable in 1 mL or g [15].

To assess the AGBS’s performance, the suitability of the method with apitoxin–royal-jelly-based anti-aging creams, propolis–honey-based toothpaste, and bee-pollen- and apitoxin–royal-jelly-based cream was tested, and it was shown to provide chemical neutralization of all of these antimicrobial agents. Once the suitability of the method was proven, calibration curves were constructed by effecting simultaneous recovery of the microorganisms by means of the RSM and the AGBS for each bee-made product using *S. aureus*, *E. coli*, *P. aeruginosa*, *C. albicans*, and *A. brasiliensis* as representatives of all TAVCs in order to demonstrate compliance with essential validation criteria, such as linearity, equivalence of the results, operative range, precision, accuracy, ruggedness, limit of detection, and limit of quantification [12].

The AGBS is based on microbial metabolism using a nutrient-based liquid medium with dextrose as the carbon source (NF-TVC vials)**.** NF-TVC vials are divided into two zones, growing and reading. Microorganisms are allowed to grow in the growing zone (liquid-enriched medium), which permits the growth of a broad spectrum of aerobic microorganisms, including bacteria, yeasts, and molds. In this way, mesophiles grow in the NF-TVC vial, yielding carbon dioxide, which diffuses through a gas-permeable layer from the growth medium toward the reading zone, which is a soft agar plug containing a dye indicator of the Soleris NF-TVC vial. Only gases can enter the reading zone; microorganisms, the medium, and particulates are blocked. When carbon dioxide reaches the reading zone of the vial, it will trigger a color change from green (the absence of microorganisms) to yellow (the presence of a microorganism) [12,13,14,15,16,17,18]. This colorimetric change is detected and recorded by the equipment’s software and corresponds to the detection time (DT, h), indicative of a positive test result [12,13,14,15,16,17,18].

## 2. Materials and Methods

### 2.1. Reagents

Apitoxin, propolis, bee pollen, and royal jelly were collected in Algeciras, Huila, Colombia (2°31′19′′ N, 75°18′52′′ W), during the January 2025 season, a region dominated by oak forests and diverse flowering species that support rich bee foraging. Apitoxin was obtained using an electrostimulation-based collection system (12 V for 45 min), following established apicultural protocols. Immediately after collection, all materials were stored and transported under controlled conditions to preserve their quality attributes. Specifically, bee venom and royal jelly were kept at 2–6 °C and 30–70% RH in light-resistant glass containers, while propolis, honey, and bee pollen were stored at 20–30 °C and 30–70% RH in tightly sealed containers. These raw bee-derived materials were used for bulk compounding of cosmetic products.

The bee-made products chosen for the present investigation, along with their antimicrobial agents, are summarized in Table 1, as described in the literature. All cosmetic products were commercial formulations provided by Laboratorios Coaspharma (Bogotá, Colombia, SensoLife Bio^®^ line). Their compositions are now explicitly listed to ensure reproducibility and clarity: (1) Anti-aging cream (SensoLife Bio^®^): Citric acid, butylated hydroxytoluene, phenoxyethanol, ethylhexyloxyphenol, solid pollen, royal jelly, and apitoxin. (2) Hair treatment (SensoLife Bio^®^): Royal jelly, phenoxyethanol, ethylhexyloxyphenol, apitoxin, cetostearyl alcohol, and glycerin. (3) Toothpaste (SensoLife Bio^®^): Propylene glycol, glycerin, polyethylene glycol, propolis, honey, and 70% non-crystallizable sorbitol.

For each bee-made product, three different lots were selected to conduct microorganism recovery for both the AGBS and the RSM. The strains *A. brasiliensis* (Cat. No. ATCC 16404), *C. albicans* (Cat. No. ATCC 10231), *P. aeruginosa* (Cat. No. ATCC 9027), *E. coli* (Cat. No. ATCC 8739), and *S. aureus* (Cat. No. ATCC 6538) were studied. Test microorganisms were from frozen stocks using culture maintenance techniques to ensure that viable microorganisms used for inoculation were not more than five passages removed from the master seed-lot. The reagents, namely tryptic soy broth, TSB (Scharlab, Barcelona, Spain, code 02-200); Sabouraud dextrose agar, SDA (Neogen, Lansing, MI, USA, cat. No. NCM0008); tryptic soy agar, TSA (Scharlab, code 02-200); and Tween^®^ 80 (Scharlab, code 73625), were used as provided.

### 2.2. Automated Growth-Based System

The Soleris^®^ 128 instruments and supplies were acquired from Neogen. This system includes four incubator drawers (128 vial places) with precise temperature control for each drawer (28.5 ± 0.5 °C), with dedicated software and a computer. The design and installation qualifications were performed by Neogen, the equipment supplier, in accordance with the vendor-specific protocols and certification reports. Operational qualification (OQ) involved verification of instrument calibration, temperature stability, and system responsiveness using standardized microbial controls. Performance qualification (PQ) was conducted by the user and consisted of verifying the Soleris system’s ability to detect microbial contamination at multiple bioburden thresholds within the defined 48 h incubation period, with the detection times corroborated by indicator color changes. NF-TVC vials containing a nutrient-based medium with dextrose as the carbon source were used to quantify *S. aureus*, *E. coli*, *P. aeruginosa*, *C. albicans*, and *A. brasiliensis*, representing the TAVCs.

### 2.3. Inoculum Standardization

*S. aureus*, *E. coli*, and *P. aeruginosa* were reactivated from frozen stocks in TSA and incubated for 48 h at 30–35 °C. *C. albicans* and *A. brasiliensis* were reactivated from frozen stocks in SDA and incubated for 48 h at 20–25 °C. After the growth time was completed, the isolated colonies were resuspended in 0.9% saline solution until they reached a McFarland standard of 2 (equivalent to 1.0 × 10^8^ CFU/mL). Regarding the limitations of applying McFarland standards to fungal conidia, we conducted a validation study to ensure accurate standardization of the *A. brasiliensis* inocula. Specifically, McFarland readings were cross-validated with spectrophotometric measurements at 580 nm, followed by serial dilution and plate count experiments to correlate optical readings with colony-forming unit (CFU) direct counts. These experiments confirmed that a McFarland 2 suspension corresponded to approximately 1.0 × 10^8^ CFU/mL for *A. brasiliensis*. The complete datasets, including dilution series and recovery counts, are provided in the Supplementary Material.

### 2.4. Suitability of the Method

In this study, polysorbate 80 (Tween^®^ 80, 0.1% *w*/*v*) was employed as the neutralizer. This compound was chosen because of its broad capacity to inactivate the preservative agents commonly found in cosmetic formulations, including parabens, alcohols, phenoxyethanol, and ethylhexyloxyphenol, as well as naturally occurring antimicrobial constituents of bee-derived products such as melittin (apitoxin), flavonoids (propolis), 10-HDA (royal jelly), and phenolic compounds (honey and bee pollen). The neutralization step was performed by incorporating polysorbate 80 into the test matrix prior to microbial inoculation, with the contact time and mixing conditions optimized to ensure homogeneity. Recovery of the test organisms (*A. brasiliensis*, *C. albicans*, *S. aureus*, *E. coli*, and *P. aeruginosa*) was then confirmed using both the AGBS and the RSM. Tween 80 (0.1% *w*/*v*) was assessed in accordance with the USP <1227> requirements, which specify the evaluation of the neutralizer’s effectiveness and toxicity and potential matrix interference. The validation protocol included the following: (1) Neutralizer effectiveness: The microorganisms (*S. aureus*, *E. coli*, *P. aeruginosa*, *C. albicans*, and *A. brasiliensis*) were spiked into preservative-containing formulations with Tween 80. Recovery was compared to that in controls without the product or the neutralizer (see the Supplementary Material). (2) Neutralizer toxicity: Organisms were inoculated into media containing only Tween 80 to confirm the absence of growth inhibition. Acceptance criteria were defined as 70–130% recovery compared with that in uninhibited controls. The complete recovery datasets and spiking experiments are presented in the Supplementary Material.

Three commercially available personal care products were tested: a hair treatment, an anti-aging cream, and a toothpaste. Each of these formulations incorporated bee-derived active ingredients—apitoxin, royal jelly, propolis, honey, and bee pollen—selected for their known biological activity and relevance to cosmetic applications. The bee-derived product’s preservatives (phenols, flavonoids, enzymes, fatty acids, melittin-peptide, and other antimicrobial agents) were neutralized using polysorbate 80 to obtain a successful microbiological determination by means of the RSM and the AGBS, to set up the equivalence between the methods and generate a calibration curve for the apitoxin–royal-jelly-based anti-aging creams, propolis–honey-based toothpaste, and bee-pollen-, apitoxin-, and royal-jelly-based creams for validation. Thus, carrying out independent assays, 1 mL of inoculum dilution from each microorganism (*S. aureus*, *E. coli*, *P. aeruginosa*, *C. albicans* and *A. brasiliensis*) was added to a Schott bottle and mixed with 90 mL of tryptic soy broth in the presence of the selected neutralizing agent (1 mL/L of Tween^®^ 80 in TSB). Then, 10 g or 10 mL of the corresponding bee-derived product was added, and it was vigorously shaken to ensure homogenization of the sample. To ensure reproducibility and capture between-lot variability, the inoculation/neutralization experiments (assessing the method’s suitability) were conducted using three independent production lots of each bee-derived product (biological replicates). For each lot, *S. aureus*, *E. coli*, *P. aeruginosa*, *C. albicans*, and *A. brasiliensis* were tested independently, and for each microorganism (*S. aureus*, *E. coli*, *P. aeruginosa*, *C. albicans*, and *A. brasiliensis*), serial dilution was performed for all strains to ensure an operational range that covered 1 CFU to approximately 1 × 10^3^ CFU. From this procedure, recovery measurements for each strain were simultaneously performed using both the AGBS and the RSM to establish the equivalence between the methods and generate a calibration curve for validation. Plate counts were performed in duplicate to reduce plating errors. This experimental design was selected to provide robust estimates of accuracy, precision, and equivalence across products and organisms.

### 2.5. Calibration Curve

Once the efficacy of the neutralizing polysorbate on the bioactive compounds from the apitoxin-, royal-jelly-, propolis-, bee-pollen-, and honey-based products had been proven, calibration curves were built by testing the 5 strains. From this, a serial dilution was performed. From each dilution (D1–D5), 1 mL was directly placed into each NF-TVC vial, and the vials were incubated for 48 h at 28.5 °C in the Soleris equipment. Simultaneously, TSA plates were inoculated in duplicate with 1 mL of each dilution using the spread plate technique. The plates were incubated at 30 °C for 7 days. This series of experiments was performed using at least three different lots for each bee-derived product. An uninoculated NF-TVC vial was used as a negative control, and an inoculated vial with 1 mL of the dilution (count of 10–100 CFU/mL) was used as a positive control for each strain tested. Each detection time (DT) recorded by the Soleris^®^ system within the incubation period (48 h), also confirmed by the color change, was an indication of the presence of microorganisms. The data generated by both the AGBS (detection times, DT) and the RSM (colony forming unit, CFU) were plotted in Soleris@ software version 7.3 to generate calibration curves by plotting the DTs relative to the corresponding log CFU values.

### 2.6. Linearity and Equivalence of Results

The plotted values in the calibration curve were fit to a least-squares regression, and the coefficient of determination (R^2^) was calculated. The linearity was determined using the goodness-of-fit test to evaluate the relationship between the CFUs and the DT data obtained from the RSM and the AGBS, respectively. In this way, for each cosmetic product tested, 5 calibration curves were constructed, corresponding to the *S. aureus*, *E. coli*, *P. aeruginosa*, *C. albicans*, and *A. brasiliensis* strains tested.

### 2.7. Accuracy

The coefficient of correlation (CC) obtained from the calibration curves was a measure of accuracy, according to USP general chapter <1223>. Additionally, the DT values and their log 10 equivalent CFU counterparts were statistically analyzed using Pearson’s goodness-of-fit test based on a Poisson distribution. Additionally, Bland–Altman plots comparing the AGBS and the RSM were generated for all strains tested as a measure of accuracy.

In this way, the DT yields with the AGBS were automatically converted into CFU by means of the calibration curves constructed for each microorganism tested. Thus, using Pearson’s goodness-of-fit test, it was possible to predict CFU from DT with a 95% confidence interval.

### 2.8. Limit of Detection and Limit of Quantification

The limit of detection (LOD) and the limit of quantification (LOQ) were determined from the calibration curves based on the fewest number of recoverable microorganisms (<10 CFU). The LOD and LOQ for both methodologies (RSM and AGBS) were calculated using the following equations: LOD = 3.3*SD/m and LOQ = 10*SD/m, where SD is the standard deviation, and m is the slope of the linear regression obtained for each calibration curve, as stated in the International Council of Harmonization (Q2) R2 and USP guidelines [19,20,21].

### 2.9. Precision

*S. aureus*, *E. coli*, *P. aeruginosa*, *C. albicans*, and *A. brasiliensis* were used to determine the suitability of the method for apitoxin–royal-jelly-based anti-aging creams, propolis–honey-based toothpaste, and bee-pollen-, apitoxin-, and royal-jelly-based creams using three different lots for each bee-derived product tested. Serial dilutions were carried out, and the microorganisms were recovered simultaneously via the RSM and the AGBS. Dilutions that recovered microorganisms in the 10 to 300 CFU range via the RSM and the AGBS were analyzed in order to determine the standard deviation (SD) and coefficient of variation (CV).

## 3. Results

### 3.1. Suitability of the Method (Antimicrobial Neutralization)

**Figure 1:**
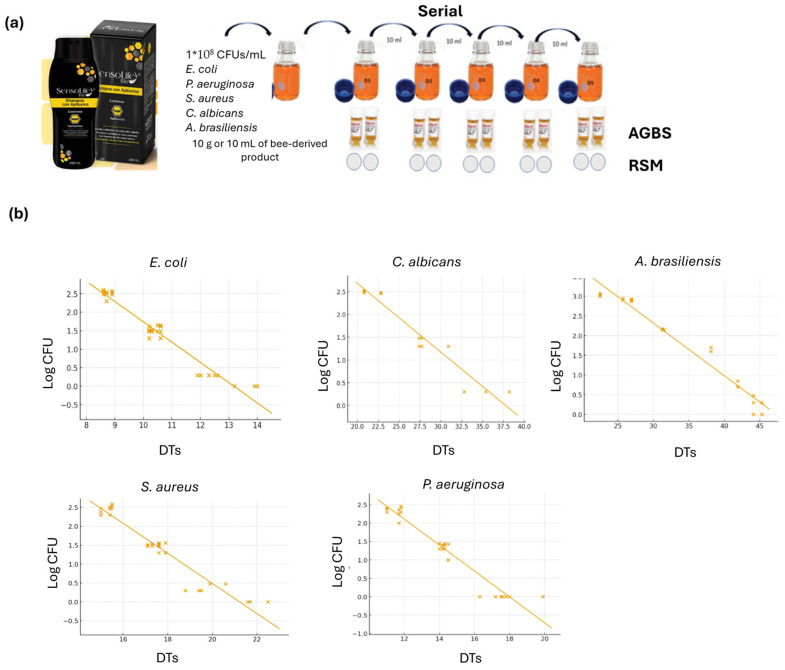
(**a**) The validation design for the automated growth-based system (AGBS) applied to bee-derived products. (**b**) Calibration curves relating detection times (DTs) to colony-forming units (CFU) for *S. aureus*, *E. coli*, *P. aeruginosa*, *C. albicans*, and *A. brasiliensis*. Bland–Altman plots show the agreement between the two measurement techniques (CFU-DTs) for all strains tested.

Antimicrobial activity has been widely reported in apitoxin, honey, royal jelly, propolis, and bee pollen due to the presence of bioactive compounds with antimicrobial properties which inhibit microbial growth [1,2,3,4,5,6,7,8,9,10,11,12] (Table 1). Hence, it is essential to consider the suppression of antimicrobial activity in all bee-made products to successfully recover microorganisms and ensure microbiological quality assessment of the product. Therefore, polysorbate 80 was used to neutralize the antimicrobial activity of the bee-derived products (Table 1). As outlined in USP <61>, polysorbate is capable of neutralizing quaternary ammonium compounds (QACs), iodine, and parabens [21]. In the current investigation, it was shown that polysorbate 80 could neutralize bioactive preservative compounds such as flavonoids, phenols, enzymes, major proteins in royal jelly, melittin, and fatty acids such as 10 HDA, permitting suitable microorganism recovery, as observed for the *S. aureus*, *E. coli*, *P. aeruginosa*, *C. albicans*, and *A. brasiliensis* calibration curves (Figure 1).

### 3.2. Linearity, Operative Range, and Equivalence of Results

Considering that the RSM and the AGBS yield quantitative data with different units (CFU vs. DT), the equivalence of the results needs to be established through building calibration curves. Thus, calibration curves for all bee-made products were derived by plotting the DT values with their respective equivalents in log CFU. The linear regression analysis yielded the relationship between the DTs and log CFU values, as shown in Table 2. The linearity observed in all bee-derived products was consistent with USP <1223> (R^2^ ≥ 0.9025), and the linearity for all bee-made products tested ranged from 1 CFU to around 1000 CFU (Table 2).

### 3.3. Accuracy

Pearson’s goodness-of-fit test was chosen to assess the comparison of the results obtained using the AGBS and those obtained using the RSM for each bee-made product for all strains tested. As shown in Table 3, the DT values can be calibrated with the CFU values according to Pearson’s goodness-of-fit test, *p* ≥ 0.05 (Table 3).

### 3.4. Limit of Detection and Limit of Quantification

The LOD and the LOQ were calculated using the standard deviation of the data obtained for the fewest number of recoverable microorganisms (<10 CFUs) and the slope of the corresponding calibration curves constructed for *S. aureus*, *E. coli*, *P. aeruginosa*, *C. albicans*, and *A. brasiliensis*. As shown in Table 4, the LODs of the AGBS for bee-pollen-, apitoxin-, and royal-jelly-based creams (capillary treatments) were 2, 3, 3, 1, and 4 CFU/mL in *S. aureus*, *E. coli*, *P. aeruginosa*, *C. albicans*, and *A. brasiliensis*, respectively, showing that it was not inferior in terms of its sensitivity compared to the RSM (<10 CFUs). Similarly, the LOD of the AGBS for the propolis–honey-based toothpaste was 1 CFU/mL for all strains tested. Finally, the LOD of the AGBS for the apitoxin–royal-jelly-based anti-aging creams was 3, 3, 1, and 4 CFUs/mL in *S. aureus*, *E. coli*, *P. aeruginosa*, *C. albicans*, and *A. brasiliensis*, respectively.

### 3.5. Intermediate Precision and Ruggedness

The precision was measured based on a dilution that recovered CFUs within a range of 10–300 using the AGBS and its equivalent in the RSM for all strains tested (*S. aureus*, *E. coli*, *P. aeruginosa C. albicans*, and *A. brasiliensis*). As shown in Table 5, for all of the bee-made products tested, the coefficient of variation (CV) was below the USP specification, <35% (30–300 CFUs).

For this experiment, the ruggedness was interpreted as the intermediate precision, a type of intra-laboratory precision that involves the effect of different lots on the test result variability, as well as repeatability (CV < 35%).

## 4. Discussion and Conclusions

While the AGBS has been broadly adopted in the food industry for semi-quantitative testing and has demonstrated utility in pharmaceutical applications for yeasts and molds, to our knowledge, it has not been systematically validated for quantitative assessment of the TAVC in bee-derived cosmetic products. Our study addresses this gap by demonstrating that the AGBS yields results equivalent to those of the compendial plate count method, meeting key USP <1223> validation criteria, including linearity, accuracy, precision, the LOD, and the LOQ. Compared to alternative rapid methods, such as ATP bioluminescence or flow cytometry, which may struggle to distinguish between viable and non-viable cells, the AGBS offers the advantage of directly monitoring microbial growth. This positions the AGBS as a scientifically robust and regulatory-aligned alternative method, with potential to expand its application beyond food and pharmaceuticals into novel cosmetic matrices. The neutralization capacity of polysorbate 80 may vary depending on the type and concentration of preservatives or bioactive compounds present in different cosmetic formulations. For this reason, suitability testing must be conducted for each product to verify both the effectiveness of the neutralizer and its lack of toxicity to the test microorganisms. This requirement represents a critical limitation of the approach, as it prevents the development of a universal, one-size-fits-all application. Nevertheless, our findings demonstrate that when properly validated, polysorbate 80 can provide reliable neutralization in bee-derived products. Future studies should explore complementary neutralizers or neutralizer combinations to broaden the applicability across diverse cosmetic matrices and ensure robust recovery in products with complex preservative systems.

We have demonstrated that polysorbate 80 can serve as a suitable chemical neutralizer of bioactive compounds with well-established antimicrobial properties, including melittin, phenols, flavonoids, enzymes, and fatty acids, such as 10-HDA. Polysorbate is a compound commonly employed in the pharmaceutical industry as a neutralizer of preservatives such as parabens, alcohols, and related antimicrobial agents. It has not been previously tested against the complex bioactive components present in bee-derived materials (e.g., melittin in apitoxin, flavonoids in propolis, 10-HDA in royal jelly, and phenolic compounds in honey and bee pollen). Our findings demonstrate that polysorbate 80 effectively neutralized these compounds under the tested conditions, allowing for reliable microbial recovery in both the automated growth-based system and the reference plate count method. By providing empirical evidence of its neutralizing capacity in these novel matrices, we strengthen the rationale for including polysorbate 80 in validation protocols for rapid microbiological methods applied to bee-derived products. Therefore, thanks to the suitability of the process, it was possible to recover an optimal microorganism from the three different bee-made matrices tested using the AGBS and the RSM. Hence, using *S. aureus*, *E. coli*, *P. aeruginosa*, *C. albicans*, and *A. brasiliensis* as suitable representatives of the TAVC, it was possible to validate essential criteria, including linearity, operational range, equivalence of the results, precision, accuracy, ruggedness, the limit of detection, and the limit of quantification.

Statistical analysis of the goodness-of-fit test demonstrated a strong relationship between the threshold bioburden (dilutions) in the NF-TVC vials and the DTs. This occurs because carbon dioxide is a direct measure of the microbial burden in the NF-TVC, so a high concentration of aerobic mesophiles in the AGBS yields low DT values, while at a low bioburden, this automated system yields high DT values.

Using Pearson’s goodness-of-fit test, the AGBS was able to predict the CFUs from the DT values with a 95% confidence interval in the calibration curves constructed for *S. aureus*, *E. coli*, *P. aeruginosa*, *C. albicans*, and *A. brasiliensis* (*p* ≥ 0.05, Table 3). Therefore, the AGBS can predict an equivalent CFU result from a DT through the calibration curve. Each tested product should have its own calibration curve since the active chemical and biological principle of the bee-made product may have a strong impact on the kinetics of the microorganism’s recovery. Although the AGBS yielded quantitative results in the DT, these results will be automatically converted into the equivalent CFU, ensuring that the AGBS has the exact specification as described by the RSM (CFU/mL).

Microbiological assessment using the RSM for TAVC detection required up to 7 days using the compendial plate count method, whereas the same results were obtained in ≤48 h using the AGBS, depending on the organism and the matrix tested.

The use of this broth-based technique (with dextrose as the carbon source) for routine analyses of bee-made products such as apitoxin–royal-jelly-based anti-aging creams, propolis–honey-based toothpaste, and bee-pollen-, apitoxin-, and royal-jelly-based creams enables the microbiological assessment to be reduced from 7 days, as is usual with the RSM, to just 2 days. This fact was clearly demonstrated by the calibration curves obtained for each bee-made product, in which the AGBS was able to detect 1 CFU/mL for the total viable counts within a maximum incubation time of 48 h, while the RSM was able to detect 1 CFU within a maximum incubation time of 7 days. The USP guidelines support the implementation of this alternative microbiological method since it provides benefits such as being less labor-intensive, reducing company warehousing costs, improving efficiency in inventory control, increasing the ability to respond more quickly to adverse microbiological results, and allowing for faster release of products onto the market, as well as being more sensitive than the RSM.

## Figures and Tables

**Table 1:** Cosmetic products tested and their respective cosmetic preservatives. For each cosmetic product, three different lots were selected.

Bee-Made Products
1. Anti-aging cream (SensoLife Bio^®^): Citric acid, butylated hydroxytoluene, phenoxyethanol, ethylhexyloxyphenol, solid pollen, royal jelly, and apitoxin.
2. Hair treatment (SensoLife Bio^®^): Royal jelly, phenoxyethanol, ethylhexyloxyphenol, apitoxin, cetostearyl alcohol, and glycerin.
3. Toothpaste (SensoLife Bio^®^): Propylene glycol, glycerin, polyethylene glycol, propolis, honey, and 70% non-crystallizable sorbitol.

**Table 2:** The linearity data obtained for the calibration curves constructed for *S. aureus*, *E. coli*, *P. aeruginosa*, *C. albicans*, and *A. brasiliensis* of each bee-made product.

Bee-Made Product	Microorganisms	Linear Regression	R^2^	x^2^ Square Test (*p* ≤ 0.05)	Upper Range of Quantification
Apitoxin–royal-jelly-based anti-aging creams	*S. aureus*	y = −2.3189x + 20.952	0.9225	*p* = 0.00	3.9 × 10^2^
	*E. coli*	y = −1.7331x + 13.046	0.9485	*p* = 0.00	4.0 × 10^2^
	*P. aeruginosa*	y = −2.6517x + 17.735	0.9343	*p* = 0.00	2.9 × 10^2^
	*C. albicans*	y = −6.1878x + 37.049	0.9319	*p* = 0.00	3.0 × 10^2^
	*A. brasiliensis*	y = −0.1605x + 7.5561	0.9692	*p* = 0.00	3.5 × 10^3^
Propolis–honey-based toothpaste	*S. aureus*	y = −2.7201x + 18.76	0.9136	*p* = 0.00	2.8 × 10^2^
	*E. coli*	y = −1.7514x + 11.9	0.9174	*p* = 0.00	2.9 × 10^2^
	*P. aeruginosa*	y = −2.89x + 17.68	0.9106	*p* = 0.00	3.0 × 10^2^
	*C. albicans*	y = −3.4748x + 25.611	0.9281	*p* = 0.00	2.0 × 10^2^
	*A. brasiliensis*	y = −0.0771x + 4.2792	0.9421	*p* = 0.00	4.0 × 10^2^
Bee-pollen-, apitoxin-, and royal-jelly-based creams (capillary treatments)	*S. aureus*	y = −2.5676x + 20.618	0.9241	*p* = 0.00	4.1 × 10^2^
	*E. coli*	y = −1.8052x + 12.46	0.9166	*p* = 0.00	5.0 × 10^2^
	*P. aeruginosa*	y = −2.5797x + 18.917	0.9204	*p* = 0.00	6.0 × 10^2^
	*C. albicans*	y = −3.6917x + 28.444	0.9206	*p* = 0.00	5.0 × 10^2^
	*A. brasiliensis*	y = −5.8226x + 41.169	0.9107	*p* = 0.00	1.0 × 10^3^

**Table 3:** Accuracy. The goodness-of-fit test (Pearson’s) and correlation coefficients obtained for all bee-derived products.

Bee-Made Product	Strains Used to Build Calibration Curves	% Recovery	Goodness-of-Fit Tests	Coefficient of Correlation
Apitoxin–royal-jelly-based anti-aging creams	*S. aureus*	81	1.0000	0.9500
	*E. coli*	100	0.9940	0.9700
	*P. aeruginosa*	102	1.0000	0.9700
	*C. albicans*	75	0.9870	0.9700
	*A. brasiliensis*	100	1.0000	0.9692
Propolis–honey-based toothpaste	*S. aureus*	103	1.0000	0.9600
	*E. coli*	100	1.0000	0.9600
	*P. aeruginosa*	107	0.9920	0.9500
	*C. albicans*	86	0.9080	0.9600
	*A. brasiliensis*	118	0.9990	0.9700
Capillary treatments	*S. aureus*	90	0.9980	0.9613
	*E. coli*	100	0.9990	0.9574
	*P. aeruginosa*	89	1.0000	0.9594
	*C. albicans*	103	0.9990	0.9595
	*A. brasiliensis*	87	1.0000	0.9543

**Table 4:** Limit of detection (LOD) and quantification (LOQ) for the RSM and the AGBS at the lowest microbial threshold before total decay in response (SD: standard deviation).

Bee-Made Products	Strains Used to Build Calibration Curves	RSM, CFU/mL			AGBS, CFU/mL		
		LOD	LOQ	SD	LOD	LOQ	SD
Apitoxin–royal-jelly-based anti-aging creams	*S. aureus*	1	3	1	2	6	1
	*E. coli*	1	3	1	3	10	2
	*P. aeruginosa*	1	1	1	1	4	1
	*C. albicans*	1	1	1	1	1	1
	*A. brasiliensis*	5	14	5	5	16	5
Propolis–honey-based toothpaste	*S. aureus*	1	1	1	1	1	1
	*E. coli*	1	3	1	4	11	2
	*P. aeruginosa*	1	2	1	3	8	2
	*C. albicans*	1	2	1	1	1	1
	*A. brasiliensis*	1	1	1	1	1	1
Bee-pollen-, apitoxin-, and royal-jelly-based cream (capillary treatments)	*S. aureus*	2	5	1	3	8	2
	*E. coli*	3	8	2	7	22	4
	*P. aeruginosa*	3	8	2	3	11	3
	*C. albicans*	1	2	1	1	3	1
	*A. brasiliensis*	4	13	4	8	24	8

**Table 5:** Precision of alternative and traditional methods for each bee-made product.

Bee-Made Product	Strains Used to Build Calibration Curves	Mean DT	Standard Deviation	Coefficient of Variation	Mean CFU	Standard Deviation	Coefficient of Variation
Apitoxin-royal jelly based anti-aging creams	*S. aureus*	17.51	0.26	1.51	30.33	5.31	17.51
	*E. coli*	10.4	0.18	1.73	33.17	8.66	26.12
	*P. aeruginosa*	19.97	3.22	16.15	21.67	6.34	29.27
	*C. albicans*	28.22	1.50	5.33	24.00	5.48	22.82
	*A. brasiliensis*	38.1	0.00	0.00	45.00	7.94	17.63
Propolis-honey based toothpaste	*S. aureus*	15.54	0.28	1.82	18.80	4.80	25.5
	*E. coli*	9.53	0.21	2.25	24.83	5.13	20.6
	*P. aeruginosa*	13.33	0.68	5.15	27.83	5.98	21.4
	*C. albicans*	20.8	0.2	0.96	21.30	2.16	10.15
	*A. brasiliensis*	39.8	0.0	0.0	31.66	2.8	9.12
Bee pollen, apitoxin, royal jelly-based shampoo	*S. aureus*	16.35	0.77	4.71	38.55	6.25	16.21
	*E. coli*	9.40	0.23	2.48	49.67	6.08	12.24
	*P. aeruginosa*	14.22	0.41	2.92	56.13	6.69	11.91
	*C. albicans*	22.42	0.80	3.61	39.86	6.01	15.08
	*A. brasiliensis*	27.70	0.00	0.00	110	10.00	9.09

## Data Availability

The original contributions presented in this study are included in the article/Supplementary Material. Further inquiries can be directed to the corresponding authors.

## Supplementary Materials

The following supporting information can be downloaded at https://www.mdpi.com/article/10.3390/microorganisms14010218/s1. Figure S1: Apitoxin–royal-jelly-based anti-aging creams; Figure S2: Propolis–honey-based toothpaste; Figure S3: Bee-pollen-, apitoxin-, and royal-jelly-based cream (capillary treatments); Table S1: Inoculum standardization; Table S2: Absorbance measurements for each test microorganism (dilutions with recovery in the range of 50–200 CFU); Table S3: Absorbance measurements performed by 3 operators; Table S4: The suitability of the method for the propolis–honey-based toothpaste; Table S5: The suitability of the method for apitoxin–royal-jelly-based anti-aging cream; Table S6: The suitability of the method for bee-pollen-, apitoxin-, and royal-jelly-based hair treatment; Table S7: Log CFU vs. DTs. Apitoxin–royal-jelly-based anti-aging creams; Table S8: Log CFU vs. DTs. Propolis–honey-based toothpaste; Table S9: Log CFU vs. DTs. Bee-pollen-, apitoxin-, and royal-jelly-based cream (capillary treatments).
