# Supplementary material for: Quantitative Assessment of Total Aerobic Viable Counts in Apitoxin-, Royal-Jelly-, Propolis-, Honey-, and Bee-Pollen-Based Products Through an Automated Growth-Based System
Source: Microorganisms. 2026 Jan 17;14(1):218. doi: 10.3390/microorganisms14010218 (PMC12843669; doi:10.3390/microorganisms14010218)
Supplement: Supplementary file 1 [file microorganisms-14-00218-s001.zip › microorganisms-3803416-supplementary.pdf]

Supplementary material

Manuscript: Quantitative assessment of total aerobic viable microorganisms in apitoxin-, royal-jelly-, propolis-, honey-, and bee-pollen-based products through an automated growth-based system

Inoculum standardization

Table S1. Inoculum standardization. **Pa** *Pseudomonas aeruginosa*; **Ab** *Aspergillus brasiliensis*; **Ca** *Candida albicans*; **Ec** *Escherichia coli*; **Sa** *Staphylococcus aureus*; **OD** optical density using McFarland standard of 2; **Abs** absorbance at 580 nm. Absorbance and optical density were verified through serial dilution and the plate count method to guarantee inoculum concentration.

	OD	Abs	CFU/mL	Log
Ec	2.03	0.69	42	1.62
	2.01	0.571	25	1.4
	2.07	0.602	52	1.72
	2.04	0.43	60	1.78
	1.99	0.599	28	1.45
	2.02	0.538	63	1.8
	2.05	0.533	60	1.78

	OD	Abs	CFU/mL	Log
Sa	2.01	0.597	23	1.36
	1.99	0.415	23	1.36
	2.06	0.541	49	1.69
	2.03	0.443	59	1.77
	2.04	0.453	34	1.53
	2.01	0.461	42	1.62
	2.04	0.486	37	1.57

	OD	Abs	CFU/mL	Log
Ca	2.04	0.706	50	1.7
	2.02	0.633	45	1.65
	2.07	0.573	42	1.62
	2.05	0.647	54	1.73
	2.06	0.535	58	1.76
	2.13	0.838	31	1.49

	OD	Abs	CFU/mL	Log
Ab	2.02	0.526	48	1.68
	2.07	0.515	55	1.74
	2.02	0.571	56	1.75
	2.07	0.573	42	1.62
	2.15	0.597	57	1.76
	2.15	0.656	32	1.51

	OD	Abs	CFU/mL	Log
Pa	2.00	0.633	30	1.48
	2.00	0.542	46	1.66
	2.08	0.536	82	1.91
	2.02	0.455	82	1.91
	1.99	0.505	39	1.59
	2.05	0.48	50	1.7
	2.01	0.425	57	1.76

Table S2. Absorbance measurements for each test microorganism (dilutions with recovery in the range of 50-200 CFU).

(a)

**Ec 10^{1}**			**Sa 10^{1}**			**Pa 10^{1}**			**Ca 10^{3}**			**Ab 10^{4}**		
**Abs**	**CFU/mL**	**Log**	**Abs**	**CFU/mL**	**Log**	**Abs**	**CFU/mL**	**Log**	**Abs**	**CFU/mL**	**Log**	**Abs**	**CFU/mL**	**Log**
0.858	50	1.7	0.773	20	1.3	0.677	70	1.85	0.697	65	1.81	1.059	88	1.94
0.859	35	1.54	0.747	40	1.6	0.65	20	1.3	0.694	58	1.76	1.059	88	1.94
0.743	55	1.74	0.626	20	1.3	0.682	43	1.63	0.699	30	1.48	1.048	98	1.99
0.752	60	1.78	0.649	42	1.62	0.658	35	1.54	0.698	26	1.41	1.032	84	1.92
0.746	61	1.79	0.669	58	1.76	0.64	85	1.93	0.681	53	1.72	1.044	89	1.95
0.881	60	1.78	0.771	46	1.66	0.716	82	1.91	0.679	97	1.99	1.031	98	1.99
0.884	55	1.74	0.744	43	1.63	0.681	52	1.72	0.685	40	1.6	1.006	98	1.99
0.756	88	1.94	0.598	40	1.6	0.625	51	1.71	0.691	96	1.98	0.942	94	1.97
0.775	39	1.59	0.607	66	1.82	0.629	65	1.81	0.663	50	1.7	0.998	97	1.99
0.849	37	1.57	0.752	34	1.53	0.621	30	1.48	0.663	50	1.7	1.081	77	1.89
0.849	37	1.57	0.752	34	1.53	0.626	54	1.73	0.665	63	1.8	0.661	59	1.77
0.755	35	1.54	0.599	36	1.56	0.626	54	1.73	0.669	72	1.86	0.65	59	1.77
0.67	30	1.48	0.550	47	1.67	0.578	56	1.75	0.666	33	1.52	0.852	52	1.72
0.563	41	1.61	0.482	47	1.67	0.532	58	1.76	0.679	70	1.85			
0.542	36	1.56	0.441	24	1.38	0.512	44	1.64	0.715	57	1.76			
0.593	52	1.2	0.495	45	1.65	0.537	58	1.76	0.673	80	1.9			
0.623	38	1.58	0.499	37	1.57	0.539	58	1.76	0.754	54	1.73			
0.543	59	1.77	0.451	76	1.88	0.477	58	1.76	0.669	96	1.98			

(b)

**Microorganisms**	**Average**	**Minimal Abs**	**Maximum Ab**
Ec	0.736	0.636	0.836
Sa	0.623	0.523	0.723
Pa	0.569	0.469	0.669
Ca	0.684	0.584	0.784
Ab	0.959	0.859	1.059

Table S3. Absorbance measurements performed by 3 operators.

(a)

Operator 1														
Ec 10^{1}			Sa 10^{1}			Pa 10^{1}			Ca 10^{3}			Ab 10^{4}		
Abs	CFU/mL	Log	Abs	CFU/mL	Log	Abs	CFU/mL	Log	Abs	CFU/mL	Log	Abs	CFU/mL	Log
0.78	50	1.7	0.629	20	1.30	0.629	46	1.66	0.677	92	1.96	1.004	54	1.73
0.78	46	1.66	0.616	40	1.60	0.603	46	1.66	0.689	66	1.82	0.938	112	2.05
0.741	47	1.67	0.619	40	1.60	0.588	42	1.62	0.686	48	1.68	1.05	125	2.10
0.757	41	1.61	0.613	41	1.61	0.589	36	1.56	0.685	49	1.69	1.014	122	2.09
0.782	40	1.6	0.612	36	1.56	0.652	30	1.48	0.697	50	1.7	0.898	20	1.3

(b)

Operator 2														
Ec 10 1			Sa 10 1			Pa 10 1			Ca 10 3			Ab 10 4		
Abs	CFU/mL	Log	Abs	CFU/mL	Log	Abs	CFU/mL	Log	Abs	CFU/mL	Log	Abs	CFU/mL	Log
0.796	70	1.85	0.696	20	1.30	0.634	98	1.99	0.636	73	1.86	0.933	23	1.36
0.753	44	1.64	0.66	44	1.64	0.627	46	1.66	0.657	74	1.87	1.054	79	1.9
0.787	47	1.67	0.653	36	1.56	0.577	60	1.78	0.672	74	1.87	1.022	83	1.92
0.745	54	1.73	0.679	44	1.64	0.564	58	1.76	0.755	84	1.92	0.955	20	1.3
0.803	80	1.9	0.564	29	1.46	0.642	78	1.89	0.598	66	1.82	1.018	27	1.43

(c)

Operator 3														
Ec 10 1			Sa 10 1			Pa 10 1			Ca 10 3			Ab 10 4		
Abs	CFU/mL	Log	Abs	CFU/mL	Log	Abs	CFU/mL	Log	Abs	CFU/mL	Log	Abs	CFU/mL	Log
0.764	35	1.54	0.658	14	1.15	0.606	42	1.62	0.67	81	1.91	1.033	118	2.07
0.79	52	1.72	0.629	59	1.77	0.644	58	1.76	0.672	72	1.86	1.056	102	20.8
0.803	39	1.59	0.643	43	1.63	0.604	50	1.7	0.69	97	1.99	0.995	32	1.51
0.668	27	1.43	0.634	12	1.08	0.603	44	1.64	0.71	45	1.65	0.918	97	1.99

Neutralizer effectiveness and toxicity and potential matrix interference

Tween 80 (0.1% w/v) was assessed in accordance with USP <1227>'s requirements, which specify the evaluation of the neutralizer's effectiveness and toxicity and potential matrix interference. The validation protocol included

1. Neutralizer effectiveness: Microorganisms (*S. aureus, E. coli, P. aeruginosa, C. albicans,* and *A. brasiliensis*) were spiked into preservative-containing formulations with Tween 80. Recovery was compared to that in controls without the product or the neutralizer (see the Supplementary Material).
2. Neutralizer toxicity: Organisms were inoculated into media containing only Tween 80 to confirm the absence of growth inhibition.

Acceptance criteria were defined as 70–130% recovery compared with that in uninhibited controls.

Prior to full method validation, we conducted a dedicated neutralizer suitability study to demonstrate that polysorbate 80 (Tween® 80, 0.1% w/v) effectively counteracts the inhibitory effects of both formulation preservatives and naturally occurring bioactives in bee-derived ingredients. Targeted inhibitory compounds included melittin (apitoxin), 10-HDA (royal jelly), flavonoids (propolis), and phenolic constituents (honey and bee pollen), as well as common synthetic preservatives (phenoxyethanol, ethylhexyloxyphenol).

The neutralization protocol was performed as follows for the three cosmetic products tested:

1. Preparation of inocula: Working suspensions (50–200 CFU/mL) of each test organism (*S. aureus, E. coli, P. aeruginosa, C. albicans,* and *A. brasiliensis*) were prepared.
2. Test sample assembly: In brief, 1 mL of inoculum was mixed with 90 mL of TSB containing Tween® 80 (0.1% w/v) in a Schott bottle, followed by the addition of 10 mL of the test product. Samples were vigorously shaken to ensure homogeneity and processed immediately.
3. Controls implemented:
   • Positive growth control: Baseline recovery was established with 1 mL inoculum + TSB (no Tween, no product).
   • Neutralizer toxicity control: It was confirmed that Tween 80 does not inhibit growth with 1 mL inoculum + TSB + Tween 80 (no product).

4. Quantitative assessment: Serial dilutions and plate counts were performed to compare recoveries.
5. Acceptance criteria: recovery within the pre-defined acceptance range (consistent with compendial practice — e.g., ≥70% of positive control) was required to deem the neutralizer effective and non-toxic.

Across all tested products and organisms, the recoveries exceeded 70% relative to that in positive controls, and no evidence of neutralizer toxicity was observed. These results indicate that Tween 80 (0.1% w/v) effectively neutralized the inhibitory activity under the tested conditions, allowing for reliable enumeration using both the reference plate count method and the AGBS.

Table S4. The suitability of the method for the propolis–honey-based toothpaste. The suitability of the method for propolis–honey-based toothpaste. **P** presence; **Pa** *Pseudomonas aeruginosa*; **Ab** *Aspergillus brasiliensis*; **Ca** *Candida albicans*; **Ec** *Escherichia coli*; **Sa** *Staphylococcus aureus*.

(a) Batch 1 and batch 2

	Propolis–honey-based toothpaste				
	1 mL inoculum + TSB, neither Tween 80 nor cosmetic product				
	**Sa**	**Pa**	**Ab**	**Ca**	**Ec**
R1	32	17	26	19	P
R2	29	22	22	21	P
R3	27	16	18	20	P
Average	29	18	22	20	P

(b)

	Propolis–honey-based toothpaste				
	1 mL inoculum + TSB + Tween 80, without cosmetic product				
	**Sa**	**Pa**	**Ab**	**Ca**	**Ec**
R1	30	15	19	20	P
R2	27	16	23	18	P
R3	31	18	20	17	P
Average	29	16	21	18	P
% Recovery	100	89	94	92	P

(c)

		Propolis–honey-based toothpaste				
		Suitability of the method (1 mL inoculum + TSB + Tween 80 + 10g cosmetic product)				
		**Sa**	**Pa**	**Ab**	**Ca**	**Ec**
**Batch 1**	R1	36	17	21	17	P
	R2	33	13	17	15	P
	R3	29	13	21	14	P
**Batch 2**	R1	31	19	18	17	P
	R2	27	18	15	15	P
	R3	20	14	19	13	P
	Average	29	16	19	15	P
	% Recovery	100	96	90	83	P

(a) Batch 3

	Propolis–honey-based toothpaste				
	1 mL inoculum + TSB, neither Tween 80 nor cosmetic product				
	**Sa**	**Pa**	**Ab**	**Ca**	**Ec**
R1	44	27	89	79	P
R2	55	33	88	89	P
R3	36	32	102	75	P
Average	45	31	93	81	P

(b)

	Propolis–honey-based toothpaste				
	1 mL inoculum + TSB + Tween 80, without cosmetic product				
	**Sa**	**Pa**	**Ab**	**Ca**	**Ec**
R1	33	20	97	71	P
R2	28	20	92	75	P
R3	45	34	87	82	P
Average	39	25	92	76	P
% Recovery	87	81	99	94	P

(c)

	Propolis–honey-based toothpaste				
	Suitability of the method (1 mL inoculum + TSB + Tween 80 + 10g cosmetic product)				
	**Sa**	**Pa**	**Ab**	**Ca**	**Ec**
R1	44	20	88	60	P
R2	41	23	73	60	P
R3	47	14	80	48	P
Average	44	19	80	56	P
% Recovery	113	76	87	74	P

Table S5. The suitability of the method for apitoxin–royal-jelly-based anti-aging cream. **P** presence; **Pa** *Pseudomonas aeruginosa*; **Ab** *Aspergillus brasiliensis*; **Ca** *Candida albicans*; **Ec** *Escherichia coli*; **Sa** *Staphylococcus aureus*.

(a) Batch 1

	Apitoxin–royal-jelly-based anti-aging cream				
	1 mL inoculum + TSB, neither Tween 80 nor cosmetic product				
	**Sa**	**Pa**	**Ab**	**Ca**	**Ec**
R1	35	32	19	18	P
R2	37	35	24	14	P
R3	38	34	19	19	P
Average	39	34	21	17	P

(b)

	Apitoxin–royal-jelly-based anti-aging cream				
	1 mL inoculum + TSB + Tween 80, without cosmetic product				
	**Sa**	**Pa**	**Ab**	**Ca**	**Ec**
R1	40	29	15	16	P
R2	37	26	20	19	P
R3	36	30	21	18	P
Average	38	28	19	18	P
% Recovery	103	84	90	104	P

(c)

	Apitoxin–royal-jelly-based anti-aging cream				
	Suitability of the method (1 mL inoculum + TSB + Tween 80 + 10g cosmetic product)				
	**Sa**	**Pa**	**Ab**	**Ca**	**Ec**
R1	29	31	17	12	P
R2	30	27	20	13	P
R3	33	24	19	14	P
Average	31	29	19	13	P
% Recovery	81	102	100	74	P

(a) Batch 2

	Apitoxin–royal-jelly-based anti-aging cream<br>1 mL inoculum + TSB, neither Tween 80 nor cosmetic product				
	**Sa**	**Pa**	**Ab**	**Ca**	**Ec**
R1	28	36	23	59	P
R2	24	38	22	62	P
R3	27	40	24	55	P
Average	26	38	23	59	P

(b)

	Apitoxin–royal-jelly-based anti-aging cream<br>1 mL inoculum + TSB + Tween 80, without cosmetic product				
	**Sa**	**Pa**	**Ab**	**Ca**	**Ec**
R1	26	40	27	69	P
R2	28	42	24	70	P
R3	25	46	25	66	P
Average	26	43	25	68	P
% Recovery	100	113	110	116	P

(c)

	Apitoxin–royal-jelly-based anti-aging cream<br>Suitability of the method (1 mL inoculum + TSB + Tween 80 + 10g cosmetic product)				
	**Sa**	**Pa**	**Ab**	**Ca**	**Ec**
R1	29	37	27	56	P
R2	31	35	34	60	P
R3	29	33	31	63	P
Average	30	35	31	60	P
% Recovery	113	82	121	87	P

(a) Batch 3

	Apitoxin–royal-jelly-based anti-aging cream				
	1 mL inoculum + TSB, neither Tween 80 nor cosmetic product				
	**Sa**	**Pa**	**Ab**	**Ca**	**Ec**
R1	22	47	21	47	P
R2	20	48	20	53	P
R3	18	42	16	52	P
Average	20	46	19	51	P

(b)

	Apitoxin–royal-jelly-based anti-aging cream				
	1 mL inoculum + TSB + Tween 80, without cosmetic product				
	**Sa**	**Pa**	**Ab**	**Ca**	**Ec**
R1	22	47	16	52	P
R2	24	43	20	52	P
R3	23	48	18	55	P
Average	23	46	18	53	P
% Recovery	115	101	95	105	P

(c)

	Apitoxin–royal-jelly-based anti-aging cream				
	Suitability of the method (1 mL inoculum + TSB + Tween 80 + 10g cosmetic product)				
	**Sa**	**Pa**	**Ab**	**Ca**	**Ec**
R1	19	47	24	53	P
R2	18	45	23	58	P
R3	15	43	20	59	P
Average	17	45	22	57	P
% Recovery	75	98	124	107	P

Table S6. The suitability of the method for bee-pollen-, apitoxin-, and royal-jelly-based hair treatment. **P** presence; **Pa** *Pseudomonas aeruginosa*; **Ab** *Aspergillus brasiliensis*; **Ca** *Candida albicans*; **Ec** *Escherichia coli*; **Sa** *Staphylococcus aureus*.

(a) Batch 1

	Bee-pollen-, apitoxin-, and royal-jelly-based cream (capillary treatments)				
	1 mL inoculum + TSB, neither Tween 80 nor cosmetic product				
	**Sa**	**Pa**	**Ab**	**Ca**	**Ec**
R1	61	39	12	63	P
R2	71	45	13	69	P
R3	85	43	13	59	P
Average	72	92	13	64	P

(b)

	Bee-pollen-, apitoxin-, and royal-jelly-based cream (capillary treatments)				
	1 mL inoculum + TSB + Tween 80, without cosmetic product				
	**Sa**	**Pa**	**Ab**	**Ca**	**Ec**
R1	53	46	16	49	P
R2	73	41	16	62	P
R3	70	55	14	70	P
Average	65	47	15	60	P
% Recovery	91	113	118	94	P

(c)

	Bee-pollen-, apitoxin-, and royal-jelly-based cream (capillary treatments)				
	Suitability of the method (1 mL inoculum + TSB + Tween 80 + 10g cosmetic product)				
	**Sa**	**Pa**	**Ab**	**Ca**	**Ec**
R1	65	38	13	66	P
R2	57	36	12	68	P
R3	54	51	13	51	P
Average	59	42	13	62	P
% Recovery	90	89	87	103	P

(a) Batch 2 and Batch 3

	Bee-pollen-, apitoxin-, and royal-jelly-based cream (capillary treatments)				
	1 mL inoculum + TSB, neither Tween 80 nor cosmetic product				
	**Sa**	**Pa**	**Ab**	**Ca**	**Ec**
R1	55	23	32	38	P
R2	64	15	30	48	P
R3	68	26	26	45	P
Average	62	21	29	44	P

(b)

	Bee-pollen-, apitoxin-, and royal-jelly-based cream (capillary treatments)				
	1 mL inoculum + TSB + Tween 80, without cosmetic product				
	**Sa**	**Pa**	**Ab**	**Ca**	**Ec**
R1	78	29	30	42	P
R2	71	30	29	36	P
R3	80	17	35	40	P
Average	76	25	31	39	P
% Recovery	122	119	105	90	P

(c)

		Bee-pollen-, apitoxin-, and royal-jelly-based cream (capillary treatments)				
		Suitability of the method (1 mL inoculum + TSB + Tween 80 + 10g cosmetic product)				
		**Sa**	**Pa**	**Ab**	**Ca**	**Ec**
**Batch 2**	R1	47	24	36	80	P
	R2	38	16	28	72	P
	R3	42	20	31	76	P
**Batch 3**	R1	70	17	29	60	P
	R2	65	20	31	52	P
	R3	65	20	30	51	P
	Average	55	20	31	65	P
	% Recovery	73	77	101	166	P

Calibration curves

Figure S1. Apitoxin–royal-jelly-based anti-aging creams.

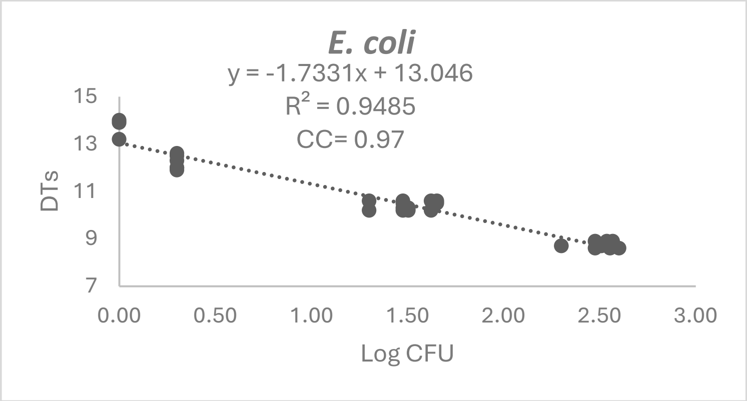

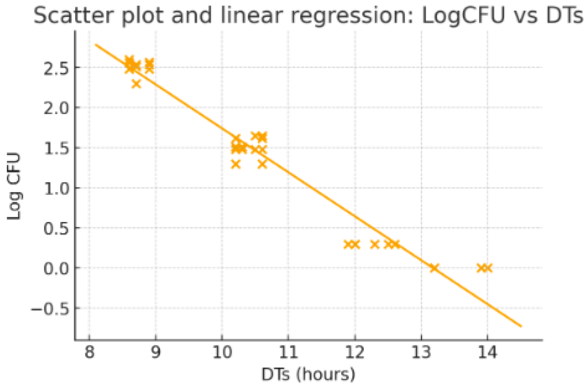

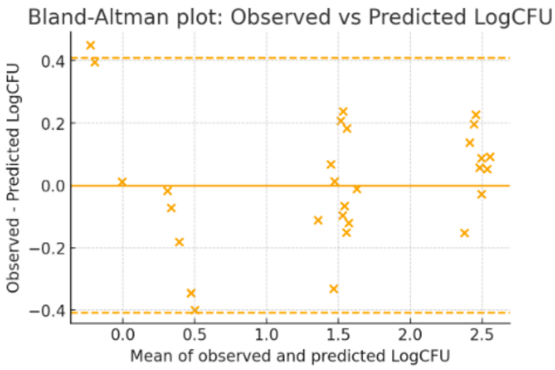

Bland–Altman comparison between observed LogCFU and LogCFU predicted from DTs showed a mean difference (bias) of 0.0000 log10 and 95% limits of agreement of −0.4090 to +0.4090 log10 (N = 30). The Shapiro–Wilk test indicated the normality of the differences (p = 0.792). No proportional bias was detected (regression of differences vs. mean: p = 0.543).

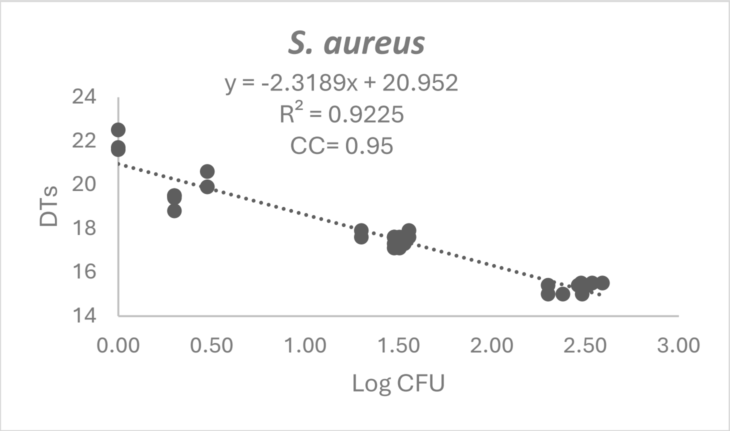

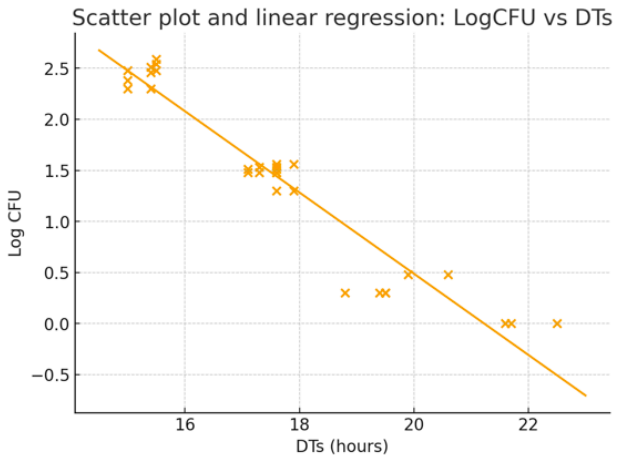

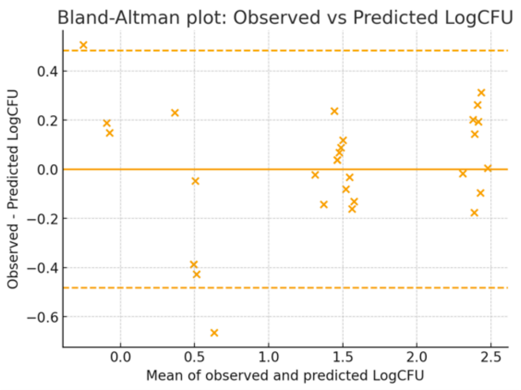

Regression equation: LogCFU = 8.4412 + (-0.3977) * DTs
Pearson's R: -0.9603
R-squared: 0.9222

P-value (slope): 4.6459986441392572e-17
Bias (mean diff): 0.0
SD of differences: 0.246

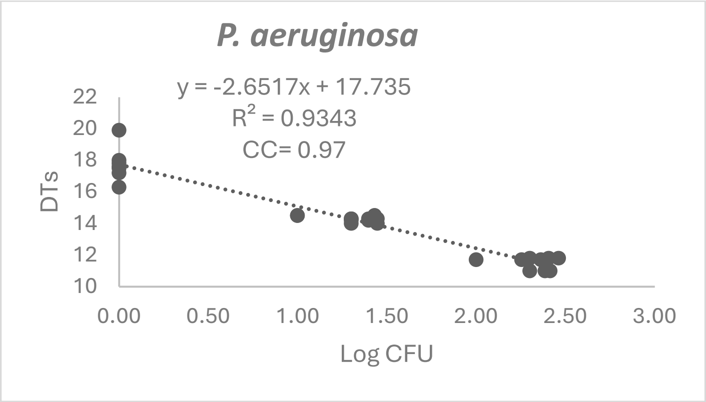

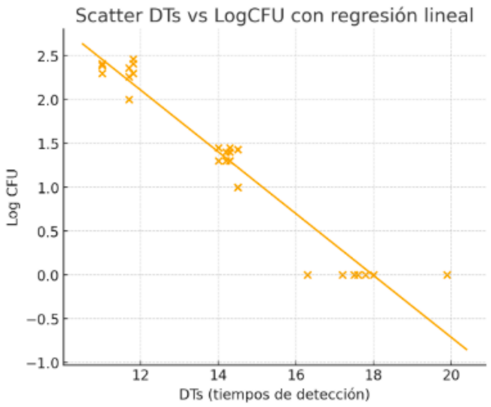

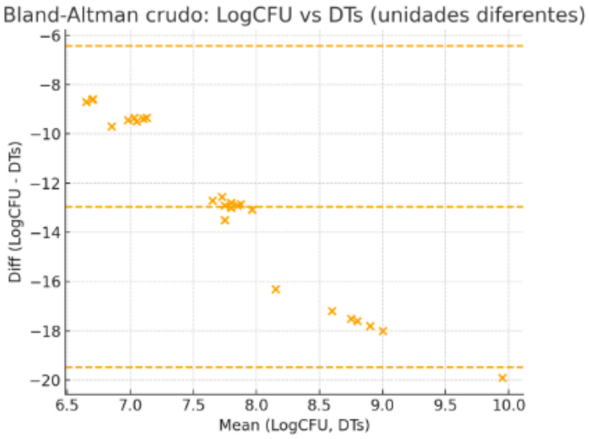

Regression equation: LogCFU = 6.3346 + (−0.3523) * DTs
Pearson's R: −0.9666
R-squared: 0.9342
P-value (slope): 6.87e−17

Bias (mean diff): 0.0

SD of differences: 0.2296

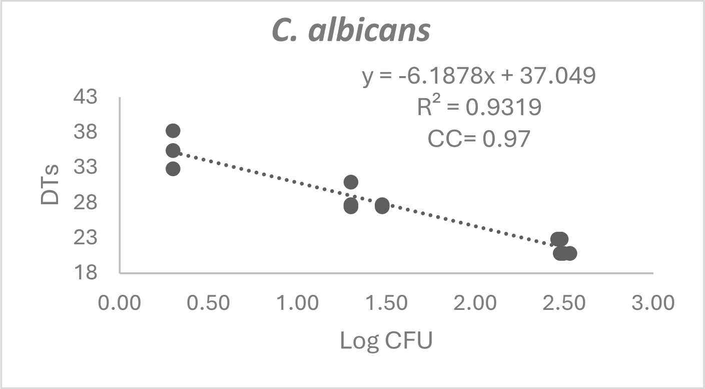

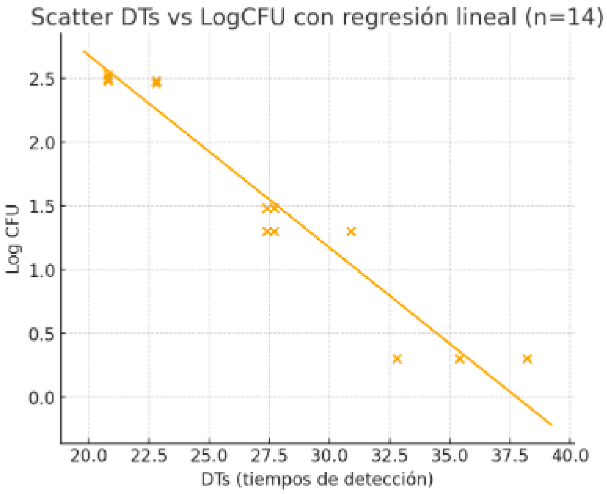

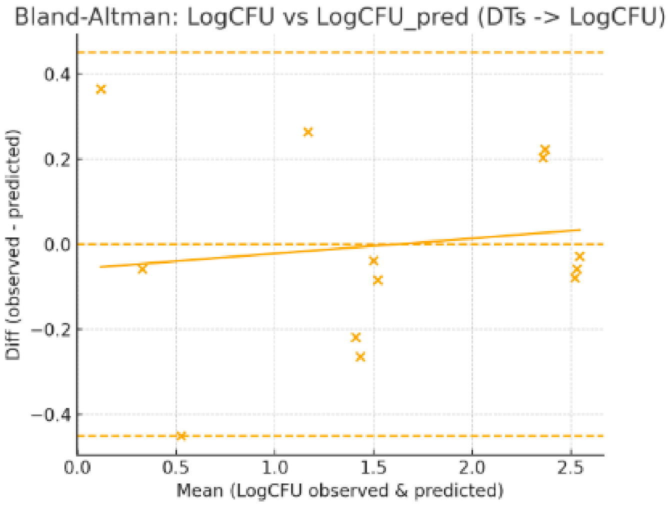

Regression equation: LogCFU = 5.6939 + (−0.1507) * DTs

Pearson’s R: −0.9655

R-squared: 0.9322

P-value (slope): 2.25e−08

Bias (mean diff): 0.0

SD of differences: 0.2295

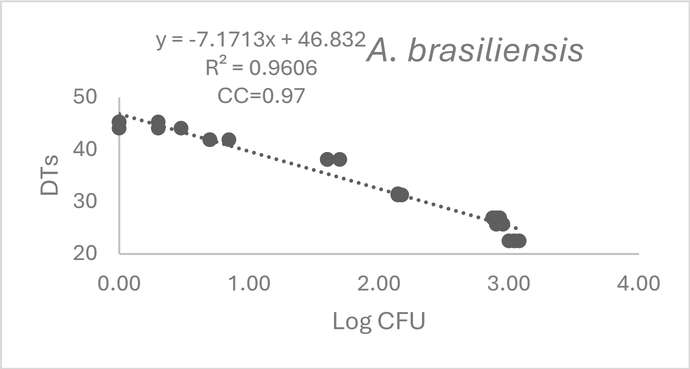

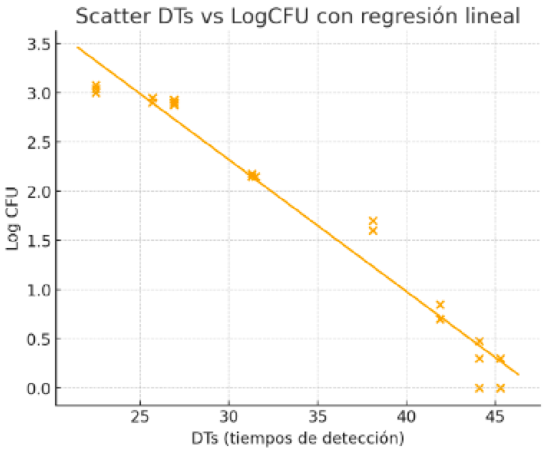

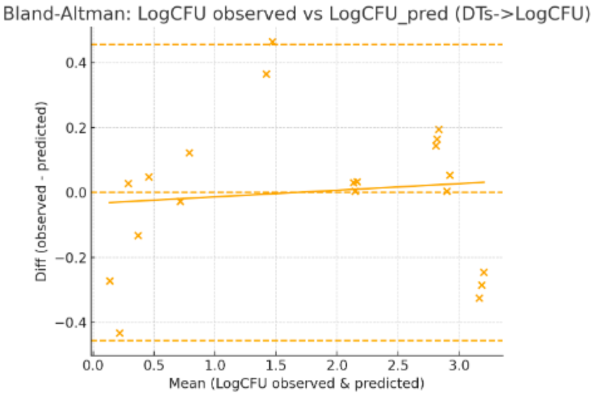

Regression equation: LogCFU = 6.3381 + (−0.1339) * DTs

Pearson’s R: −0.9801

R-squared: 0.9606

P-value (slope): 3.16e−16

Bias (mean diff): 0.0
SD of differences: 0.2325

Calibration curves

Figure S2. Propolis–honey-based toothpaste.

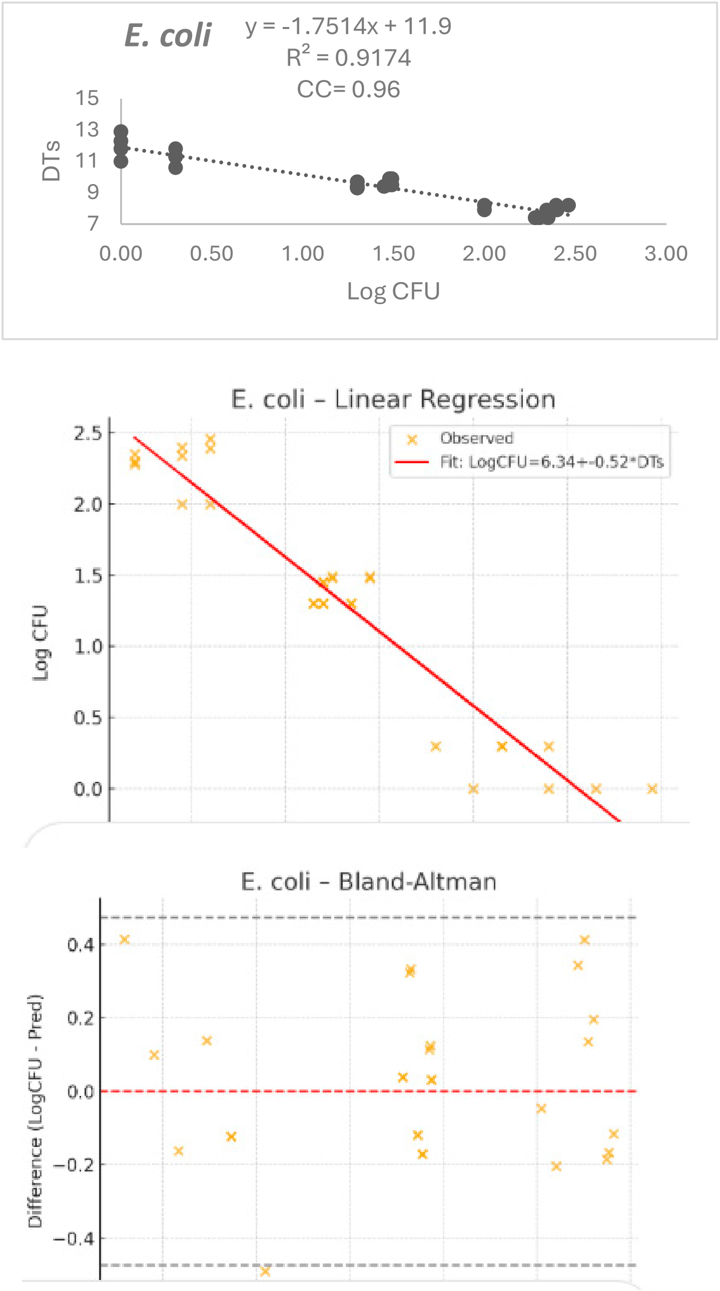

Regression equation: LogCFU = 6.34 – 0.52·DTs
Pearson's R: –0.958

R-squared: 0.917
P-value (slope): <0.0001
Bias (mean diff): –0.00
SD of differences: 0.242

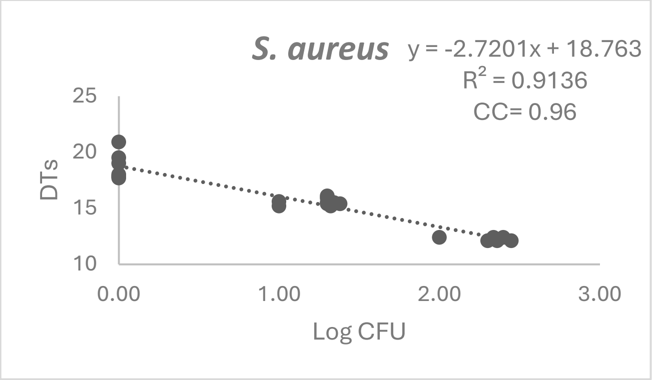

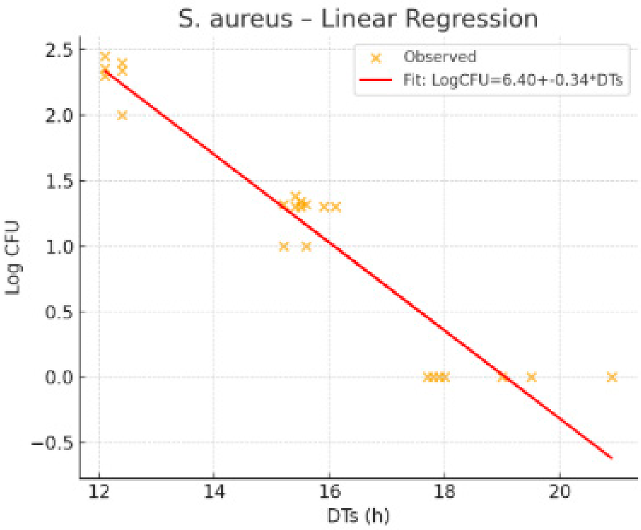

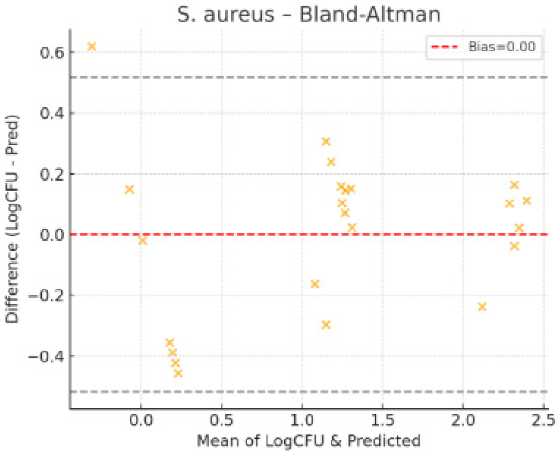

Regression equation: LogCFU = 6.40 – 0.34·DTs
Pearson's R: –0.956

R-squared: 0.914

P-value (slope): <0.0001

Bias (mean diff): 0.00

SD of differences: 0.264

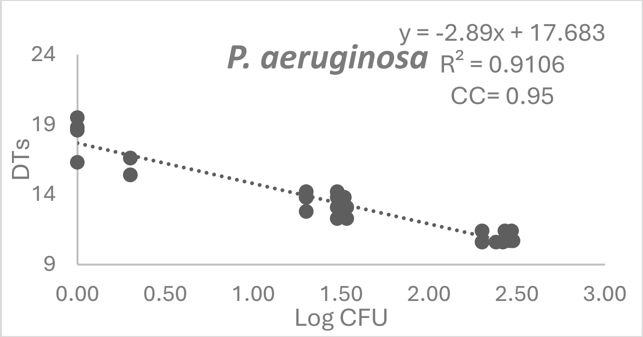

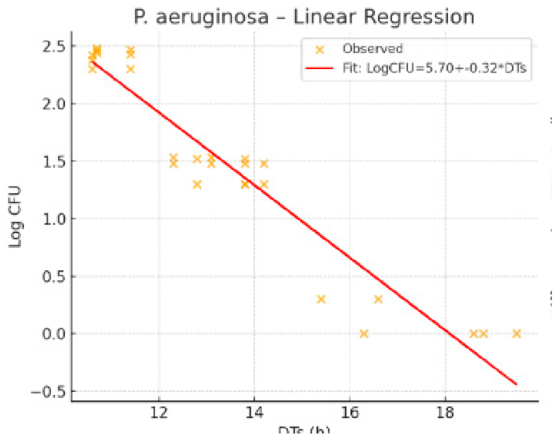

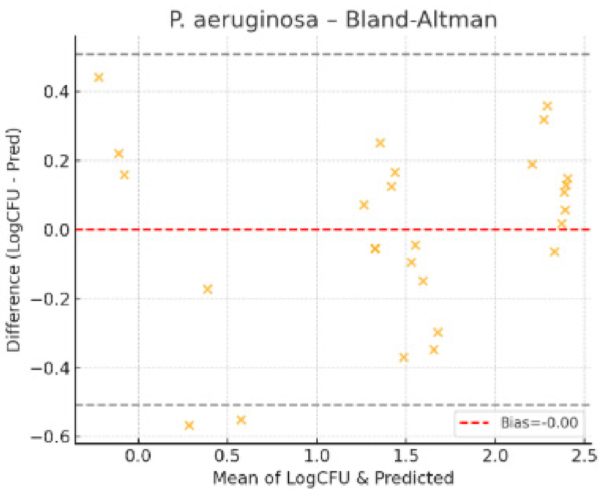

Regression equation: LogCFU = 5.70 – 0.32·DTs

Pearson's R: –0.954

R-squared: 0.910

P-value (slope): <0.0001

Bias (mean diff): –0.00

SD of differences: 0.259

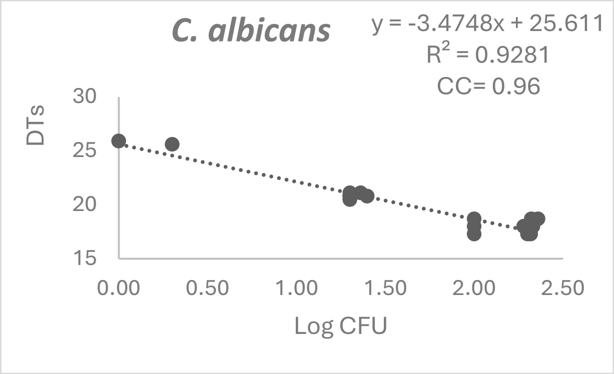

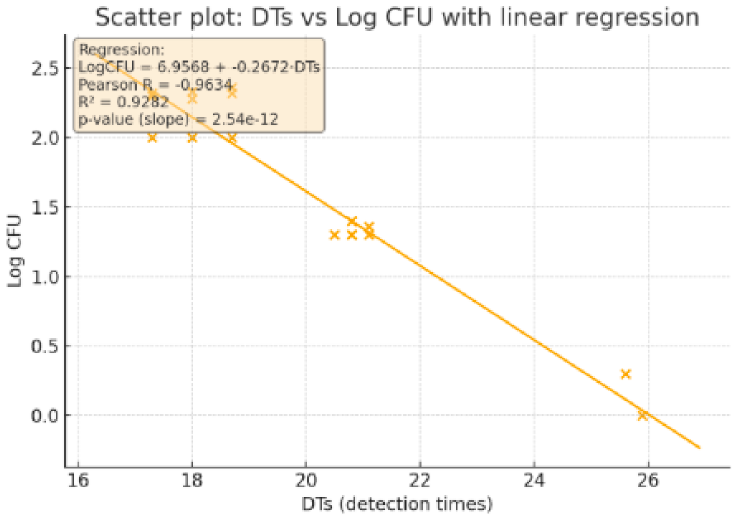

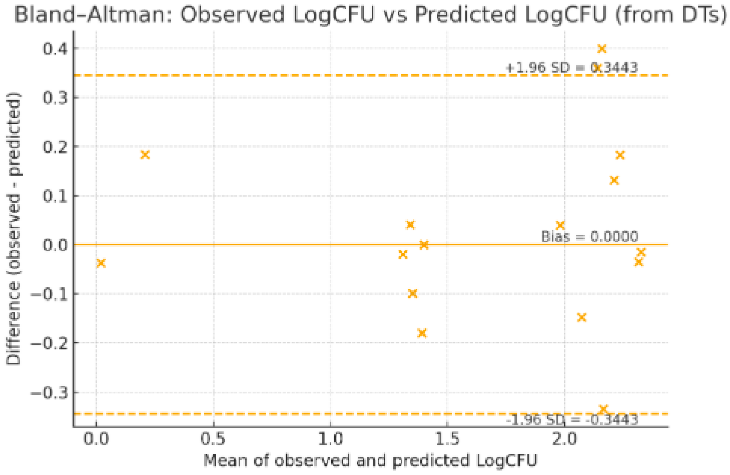

Regression equation: LogCFU = 6.9568 + -0.2672 * DTs

Pearson's R: -0.9634

R-squared: 0.9282

P-value (slope): 2.54e-12

Bias (mean diff): 0.0000

SD of differences: 0.1757

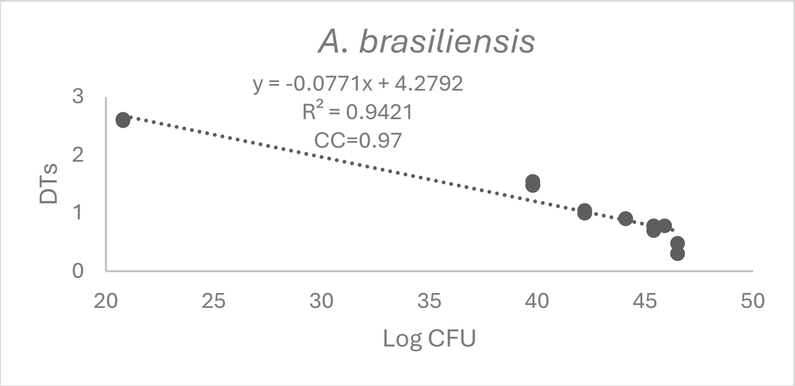

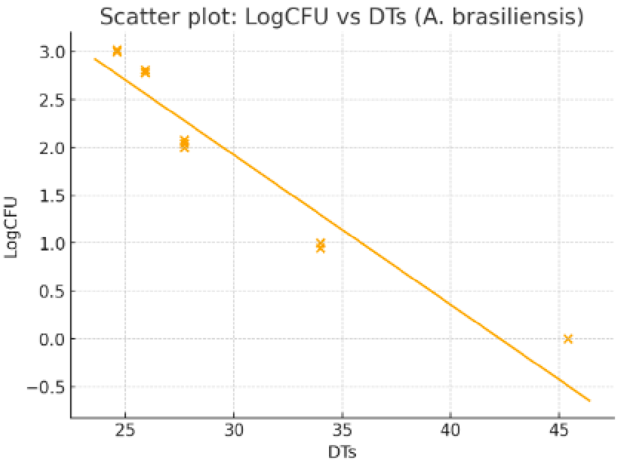

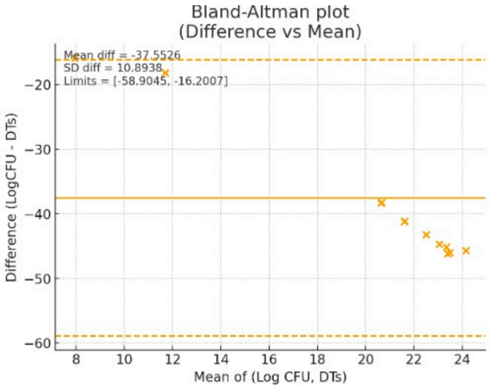

Regression equation: LogCFU = 6.6143 + -0.1564 * DTs
Pearson's R: -0.9542
R-squared: 0.9105
P-value (slope): 1.4695e-06
Bias (mean diff): -27.8283
SD of differences: 7.0598

Calibration curves

Figure S3. Bee-pollen-, apitoxin-, and royal-jelly-based cream (capillary treatments).

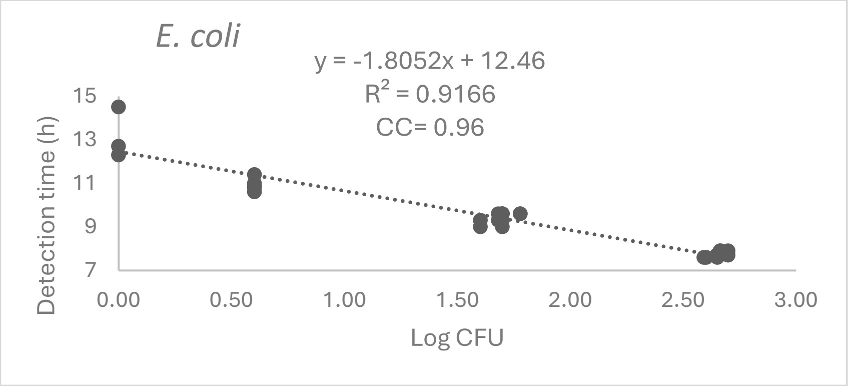

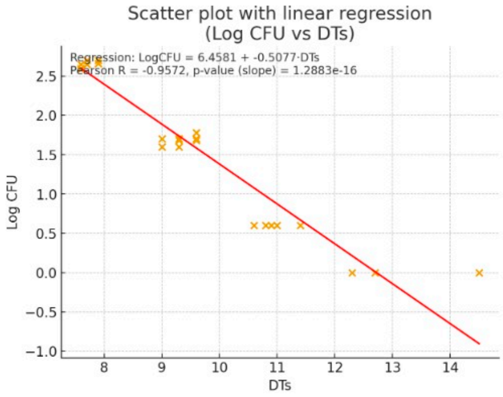

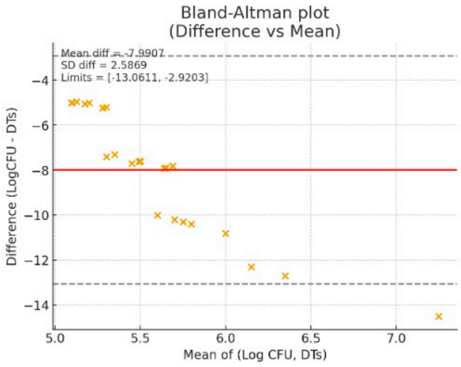

Regression equation: LogCFU = 6.458144 + -0.507702·DTs
Pearson's R: -0.957238
R-squared: 0.916304
P-value (slope): 1.288338e-16
Bias (mean diff): -7.990667
SD of differences: 2.586942

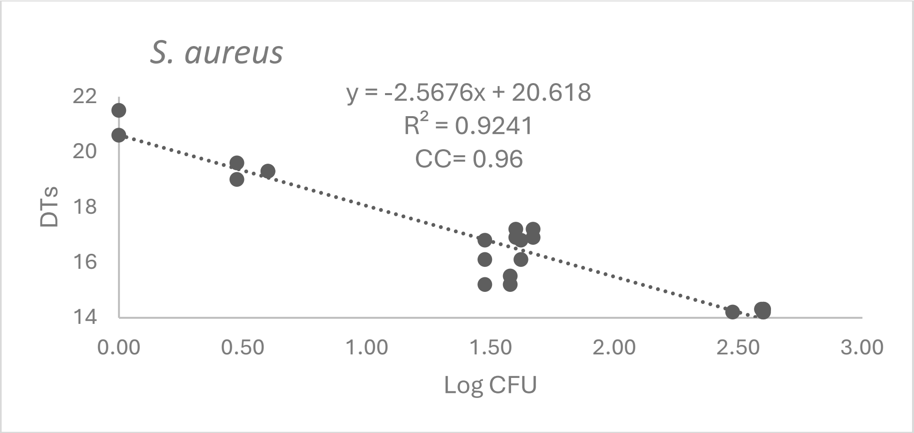

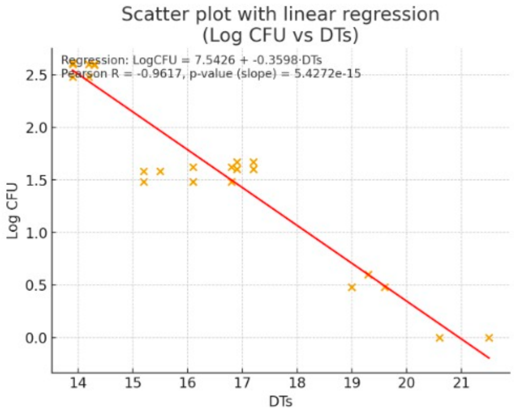

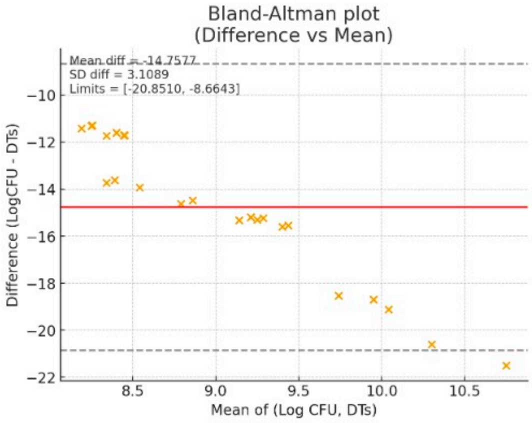

Regression equation: LogCFU = 7.542597 + -0.359774·DTs

Pearson's R: -0.961687
R-squared: 0.924843
P-value (slope): 5.427238e-15
Bias (mean diff): -14.757692
SD of differences: 3.108851

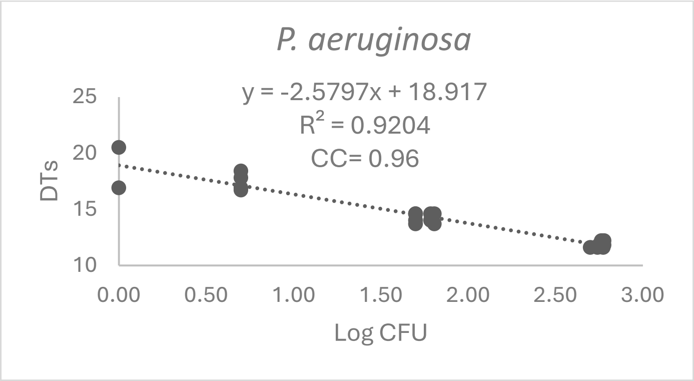

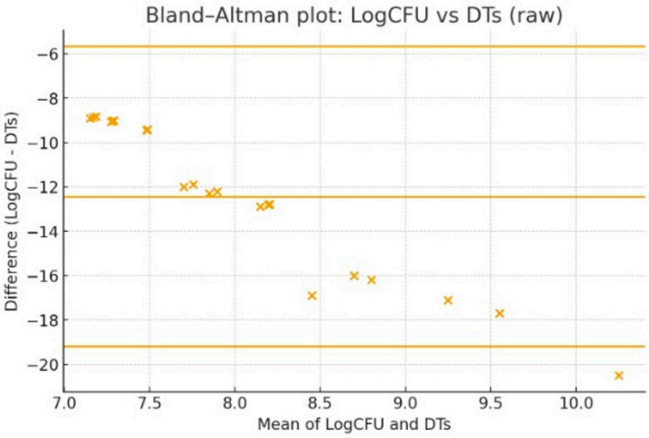

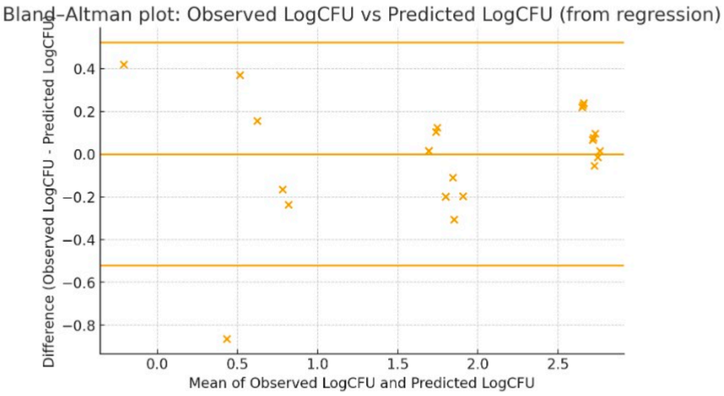

Regression equation: LogCFU = 6.8921 + (-0.3566) * DTs
Pearson's R: -0.9594
R-squared: 0.9205
P-value (slope): 5.1077e-13
Bias (mean diff): 0.0000
SD of differences: 0.2662

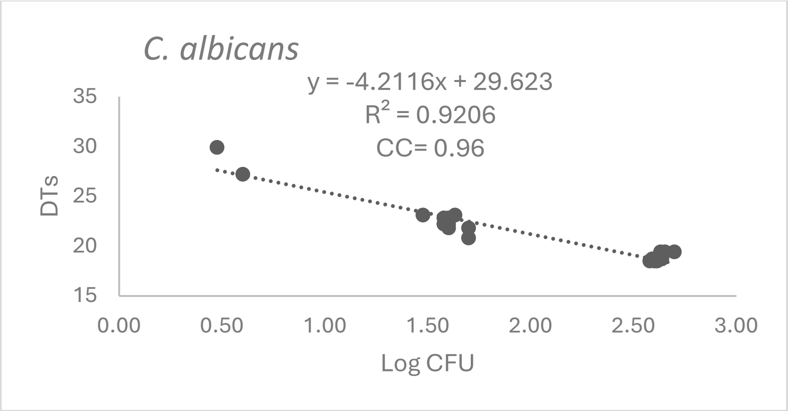

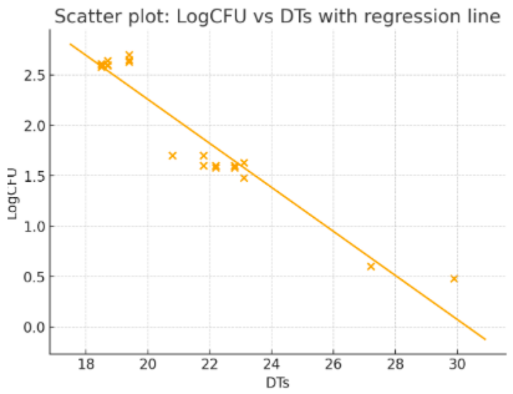

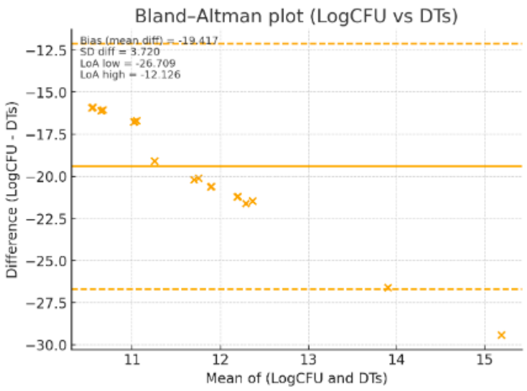

Regression equation: LogCFU = 6.6249 + -0.2184 * DTs
Pearson's R: -0.9594
R-squared: 0.9205
P-value (slope): 2.4546e-11

Bias (mean diff): -19.4175

SD of differences: 3.7200

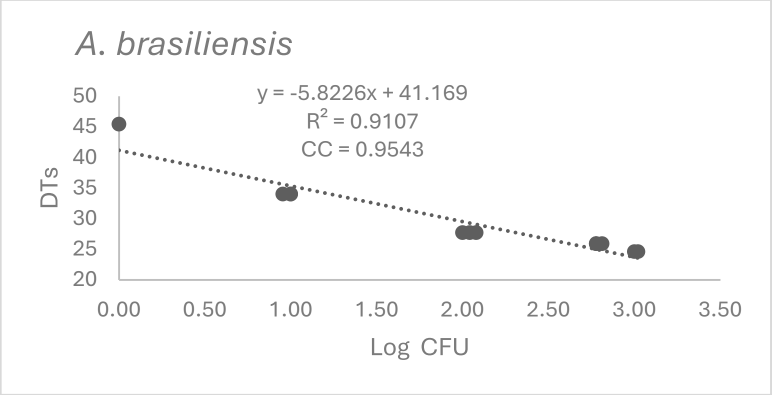

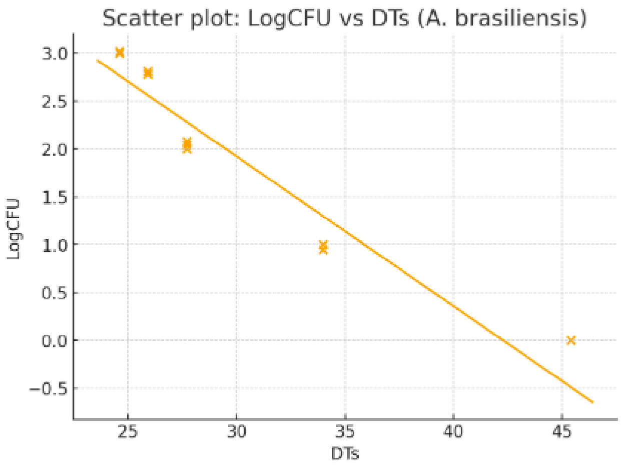

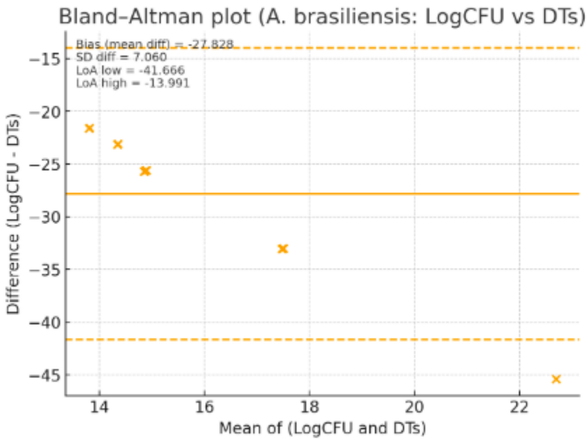

Regression equation: LogCFU = 6.6143 + -0.1564 * DTs
Pearson's R: -0.9542
R-squared: 0.9105
P-value (slope): 1.4695e-06
Bias (mean diff): -27.8283
SD of differences: 7.0598

Table S7. Log CFU vs. DTs. Apitoxin–royal-jelly-based anti-aging creams. **Pa** *Pseudomonas aeruginosa*; **Ab** *Aspergillus brasiliensis*; **Ca** *Candida albicans*; **Ec** *Escherichia coli*; **Sa** *Staphylococcus aureus*.

Apitoxin–royal-jelly-based anti-aging creams									
**Ec**		**Sa**		**Pa**		**Ca**		**Ab**	
Log UFC	DTs	Log UFC	DTs	Log UFC	DTs	2.48	22.8	0.70	41.9
2.56	8.6	2.51	15.4	2.36	11.7	2.46	22.8	0.85	41.9
2.60	8.6	2.46	15.4	2.26	11.7	2.48	22.8	0.70	41.9
2.48	8.6	2.30	15.4	2.00	11.7	1.48	27.4	0.00	45.3
1.51	10.2	1.51	17.6	1.45	14.3	1.30	27.4	0.00	45.3
1.48	10.2	1.48	17.6	1.30	14.3	1.48	27.7	0.30	45.3
0.30	12	0.30	19.5	0.00	17.2	1.30	27.7	1.70	38.1
0.00	13.2	0.00	22.5	1.45	14	0.30	35.4	1.60	38.1
1.51	10.3	1.51	17.1	1.30	14	2.50	20.8	1.60	38.1
1.48	10.3	1.48	17.1	0.00	16.3	2.53	20.8	0.48	44.1
0.30	12	0.30	18.8	2.41	11.8	2.48	20.8	0.30	44.1
2.51	8.7	2.48	15	2.46	11.8	1.30	30.9	0.00	44.1
2.54	8.7	2.38	15	2.30	11.8	0.30	32.8	3.04	22.5
2.30	8.7	2.30	15	1.43	14.5	0.30	38.2	3.00	22.5
1.65	10.5	1.56	17.9	1.00	14.5	2.48	22.8	3.08	22.5
1.48	10.5	1.30	17.9	0.00	17.8	2.46	22.8	2.90	26.9
0.30	12.6	0.30	19.4	0.00	19.9	2.48	22.8	2.88	26.9
0.00	13.9	0.00	21.6	1.43	14.5	1.48	27.4	2.93	26.9
1.65	10.6	1.56	17.6	1.00	14.5	1.30	27.4	2.95	25.7
1.48	10.6	1.30	17.6	0.00	17.6	1.48	27.7	2.90	25.7
0.30	12.3	0.30	19.5	2.39	11	1.30	27.7	2.15	31.5
2.54	8.9	2.54	15.5	2.41	11	0.30	35.4	2.15	31.3
2.57	8.9	2.59	15.5	2.30	11	2.50	20.8	2.18	31.3
2.48	8.9	2.48	15.5	1.40	14.2	2.53	20.8		
1.62	10.6	1.53	17.3	1.30	14.2	2.48	20.8		
1.30	10.6	1.48	17.3	0.00	17.5	1.30	30.9		
0.30	12.5	0.48	20.6	1.40	14.3	0.30	32.8		
0.00	14	0.00	21.7	1.30	14.3	0.30	38.2		
1.62	10.2	1.53	17.6	0.00	18				

1.30	10.2	1.48	17.6
0.30	11.9	0.48	19.9

Table S8. Log CFU vs. DTs. Propolis–honey-based toothpaste. **Pa** *Pseudomonas aeruginosa*; **Ab** *Aspergillus brasiliensis*; **Ca** *Candida albicans*; **Ec** *Escherichia coli*; **Sa** *Staphylococcus aureus*.

Ec		Sa		Pa		Ca		Ab	
**Log UFC**	**DTs**	**Log UFC**	**DTs**	**Log UFC**	**DTs**	**Log UFC**	**DTs**	**Log UFC**	**DTs**
2.35	7.4	2.34	12.4	2.44	10.7	2.33	18	1.00	42.2
2.28	7.4	2.40	12.4	2.46	10.7	2.28	18	1.04	42.2
2.30	7.4	2.00	12.4	2.48	10.7	2.00	18	1.00	42.2
1.30	9.7	1.30	15.9	1.52	13.8	1.36	21.1	0.90	44.1
1.30	9.7	0.00	19	1.30	13.8	1.30	21.1	0.90	44.1
0.30	10.6	0.00	20.9	0.30	16.6	2.32	17.3	0.70	45.4
0.00	12.3	1.38	15.4	0.00	18.8	2.30	17.3	0.70	45.4
1.30	9.3	1.30	15.4	1.52	12.8	2.00	17.3	1.48	39.8
1.30	9.3	0.00	17.9	1.30	12.8	1.30	20.8	1.48	39.8
0.30	11.3	2.36	12.1	2.47	11.4	1.30	20.8	1.54	39.8
2.40	7.9	2.45	12.1	2.43	11.4	1.30	20.5	1.30	47
2.34	7.9	2.30	12.1	2.30	11.4	1.30	20.5	1.30	47
2.00	7.9	1.32	15.6	1.48	14.2	0.00	25.9	0.00	15.9
1.45	9.4	1.00	15.6	1.30	14.2	2.36	18.7	2.59	20.8
1.30	9.4	0.00	19.5	0.30	15.4	2.32	18.7	2.61	20.8
0.00	11.8	1.32	15.2	0.00	18.6	2.00	18.7	2.60	20.8
1.45	9.4	1.00	15.2	1.48	13.8	1.40	20.8	0.48	46.5
1.30	9.4	0.00	17.8	1.30	13.8	1.30	20.8	0.30	46.5
0.00	11	1.34	15.5	0.00	16.3	1.40	20.8	0.78	45.9
2.39	8.2	1.30	15.5	2.42	10.6	1.30	20.8		
2.46	8.2	0.00	17.7	2.38	10.6	0.30	25.6		
2.00	8.2	1.30	16.1	2.30	10.6				
1.49	9.9	0.00	18	1.53	12.3				
1.48	9.9			1.48	12.3				
0.30	11.8			0.00	19.5				
0.00	12.9			1.53	13.1				
1.49	9.5			1.48	13.1				
1.48	9.5								
0.30	11.3								

Table S9. Log CFU vs. DTs. Bee-pollen-, apitoxin-, and royal-jelly-based cream (capillary treatments). **Pa** *Pseudomonas aeruginosa*; **Ab** *Aspergillus brasiliensis*; **Ca** *Candida albicans*; **Ec** *Escherichia coli*; **Sa** *Staphylococcus aureus*.

Ec		Sa		Pa		Ca		Ab	
Log UFC	DTs	Log UFC	DTs	Log UFC	DTs	Log UFC	DTs	Log UFC	DTs
2.65	7.6	2.59	14.3	2.76	12.2	2.61	18.5	1.00	34
2.59	7.6	2.60	14.3	2.77	12.2	2.58	18.5	0.95	34
2.60	7.6	2.60	14.3	2.78	12.2	2.60	18.5	1.00	34
1.68	9.3	1.58	15.2	1.79	14	1.63	23.1	2.78	25.9
1.70	9.3	1.48	15.2	1.70	14	1.48	23.1	2.81	25.9
0.60	10.6	1.58	15.5	0.70	18.4	2.63	19.4	2.78	25.9
0.00	12.7	2.60	13.9	0.00	16.9	2.65	19.4	0.00	45.4
1.68	9.6	2.61	13.9	1.79	14.6	2.70	19.4	2.00	27.7
1.70	9.6	2.48	13.9	1.70	14.6	1.70	20.8	2.08	27.7
0.60	11.4	1.62	16.8	0.70	17.8	0.60	27.2	2.04	27.7
2.66	7.9	1.48	16.8	2.76	11.8	1.70	21.8	3.00	24.6
2.66	7.9	0.60	19.3	2.75	11.8	1.60	21.8	3.02	24.6
2.70	7.9	0.00	21.5	2.78	11.8	2.64	18.7	1.00	34
1.70	9.3	1.62	16.1	2.77	11.6	2.59	18.7	0.95	34
1.60	9.3	1.48	16.1	2.74	11.6	2.60	18.7	1.00	34
0.60	10.9	0.60	19.3	2.70	11.6	1.58	22.2	2.78	25.9
0.00	14.5	2.60	14.2	1.81	13.7	1.60	22.2	2.81	25.9
1.70	9	2.60	14.2	1.70	13.7	0.48	29.9	2.78	25.9
1.60	9	2.48	14.2	0.70	16.9	1.58	22.8	0.00	45.4
0.60	10.9	1.67	17.2	0.00	20.5	1.60	22.8	2.00	27.7
2.65	7.7	1.60	17.2	1.81	14.6			2.08	27.7
2.65	7.7	0.48	19.6	1.70	14.6			2.04	27.7
2.70	7.7	0.00	20.6	0.70	16.7			3.00	24.6
1.70	9.6	1.67	16.9					3.02	24.6
1.78	9.6	1.60	16.9						
0.60	10.8	0.48	19						
0.00	12.3								
1.70	9.6								
1.78	9.6								
0.60	11								
